# Scale‐up and Sustainability Evaluation of Biopolymer Production from Citrus Waste Offering Carbon Capture and Utilisation Pathway

**DOI:** 10.1002/open.201900015

**Published:** 2019-03-07

**Authors:** Alex Durkin, Ivan Taptygin, Qingyuan Kong, Mohamad F. M. Gunam Resul, Abdul Rehman, Ana M. L. Fernández, Adam P. Harvey, Nilay Shah, Miao Guo

**Affiliations:** ^1^ Department of Chemical Engineering Imperial College London London SW7 2AZ UK; ^2^ School of Engineering Newcastle University Newcastle upon Tyne NE1 7RU UK

**Keywords:** biopolymer, copolymerisation, resource recovery, carbon dioxide fixation, life cycle analysis

## Abstract

Poly(limonene carbonate) (PLC) has been highlighted as an attractive substitute to petroleum derived plastics, due to its utilisation of CO_2_ and bio‐based limonene as feedstocks, offering an effective carbon capture and utilisation pathway. Our study investigates the techno‐economic viability and environmental sustainability of a novel process to produce PLC from citrus waste derived limonene, coupled with an anaerobic digestion process to enable energy cogeneration and waste recovery maximisation. Computational process design was integrated with a life cycle assessment to identify the sustainability improvement opportunities. PLC production was found to be economically viable, assuming sufficient citrus waste is supplied to the process, and environmentally preferable to polystyrene (PS) in various impact categories including climate change. However, it exhibited greater environmental burdens than PS across other impact categories, although the environmental performance could be improved with a waste recovery system, at the cost of a process design shift towards energy generation. Finally, our study quantified the potential contribution of PLC to mitigating the escape of atmospheric CO_2_ concentration from the planetary boundary. We emphasise the importance of a holistic approach to process design and highlight the potential impacts of biopolymers, which is instrumental in solving environmental problems facing the plastic industry and building a sustainable circular economy.

## 1 Introduction

*Background*. Global demand for plastic has increased considerably in the past decades, with the average annual increase in global plastic production reaching 10 % (from 1.5 million tonnes in 1950 to 335 million tonnes in 2016).^1^ Polystyrene (PS) is one of the most dominant distinct plastic groups with an average compound annual growth rate of 5 %. However, despite rapidly increasing plastic waste collection (by 79 %) and energy recovery (by 61 %) in the past decade, large quantities of post‐consumer plastic waste are treated in landfill, causing environmental concerns.

The depletion of fossil resources, coupled with an increasing demand for energy and plastic, have catalysed research on alternative pathways to source these products. Consequently, the utilisation of waste biomass, which is both an abundant and renewable resource, is becoming an important field of research.^2^ Sourcing value‐added bioplastics from wastes also plays an important role in the emerging circular economy, not only achieving enhanced resource efficiency but also reducing the carbon footprint compared to traditional polymer production pathways.

This study focuses on a novel biopolymer production pathway, based on the copolymerisation of carbon dioxide (CO_2_) and limonene oxide sourced from citrus waste. By up‐cycling CO_2_ (e. g. power plant flue gas) as a reagent, the biopolymer production represents a sustainable pathway which could help to mitigate climate change.^3^

Specifically, citrus waste contains d‐limonene, which can be used as a versatile feedstock for biopolymer production. As one of the most common terpenes, limonene constitutes 90–96 % of citrus peel oil.^4^ It can be oxidised to produce limonene oxide, which can then be co‐polymerised with CO_2_ to produce poly(limonene carbonate) (PLC). The feasibility of PLC production from limonene oxide was first confirmed in 2004,^4^ and since then considerable research has been published on this topic.

PLC has properties similar to PS,^5^, ^6^ making it a candidate to substitute traditional petroleum‐based polymers. Realising this paradigm shift, requires industrial‐scale process design, as initially pioneered by Dalaeli, Fogle and Yang in their feasibility study.^5^ The study uncovered a two‐part process to carry out the biopolymer production. Firstly, orange peel derived limonene was oxidised with tert‐butyl hydroperoxide (TBHP) to produce limonene oxide and tert‐butyl alcohol (TBA). Limonene oxide was then co‐polymerised with CO_2_, in the presence of a β‐diiminate zinc acetate complex catalyst,^4^ to produce PLC. Maximum turnover frequency (TOF) of 37 h^−1^ was reported at the copolymerisation conditions of 25 °C and 7 bar.^4^ The economic appraisal discovered that the levelised cost of PLC was 31 % higher than conventionally produced PS. The process was therefore deemed unfeasible unless the higher price was validated by PLC's then undiscovered properties. Furthermore, it was suggested that the PLC sale price could be reduced by optimising the overall process and finding an alternative epoxidation pathway, which was the largest contributor to the PLC high cost due to large raw material costs.

PLC′s high sale price was justified by Hauenstein et al.,^6^ encouraging further optimisation of the production process. It was found that PLC can be modified to give it properties such as hydrophilicity, pH‐dependent solubility and antibacterial properties. Additionally, PLC was converted from a thermoplastic into a rubbery material through addition of a thiol group. This extends the spectrum of PLC's possible uses after production and can be used to justify the higher sale price. Hauenstein et al. also observed the highest reported experimental TOF to date (70 h^−1^) for the copolymerisation reaction, at 20 °C and 11 bar.^6^

The environmental and economic aspects of the PLC production process were explored recently via a process design in conjunction with a LCA, which made several interesting findings.^7^, ^8^ First, a large portion of negative environmental impacts were caused by an energy intensive separation sequence, suggesting that the process could be improved by reducing the number of components in the system. Second, an alternative oxidising agent to TBHP was recommended, since the synthesis route of TBHP has a large negative environmental impact. Third, the separation sequences should be rigorously optimised to reduce the heating duties in distillation columns. Finally, it is important to recognise that the biowaste feedstock could be used as an energy source as well as a raw material for the process.^9^

Recent studies on limonene oxide reaction pathways have focused on epoxidation with hydrogen peroxide (H_2_O_2_) due to its perceived environmental benefits (producing water as a by‐product). The novel epoxidation process modelled in our study is a polytungstophosphate catalysed and solvent‐free reaction, demonstrating 100 % selectivity and reduced reaction time of 15 minutes.^10^ Such an empirical advance offers a potential sustainable solution: first, it solves the problem with expensive process feedstocks highlighted in previous studies; second, it enables environmental impact reduction by no additional solvent input since excess limonene acts as a solvent, mitigating exothermicity and allowing for higher selectivity. The former is due to the decreased overall flowrate, reducing heating duties in separation sequences and electricity use associated with pumping requirements. The kinetic data reported for the epoxidation reaction allowed new research on modelling and sizing of the reactor in this study. However, the techno‐economic potential and environmental implications of switching to this novel synthesis pathway still remain unexplored.

The global citrus fruit processing industries produce vast amounts of waste, from which limonene can be extracted and utilised in the PLC production process. More than 50 wt % of fresh oranges end up as peels and other forms of waste during orange juice production and are currently used as biomass for fertilisers or for animal feed.^11^ Production of PLC has the potential to impact the current citrus industries by recovering value‐added products from citrus waste, contributing to a circular economy and potentially improving the overall sustainability of the plastic industry. The vast amounts of waste justify consideration of anaerobic digestion (AD) and combined heat and power (CHP) integration for energy cogeneration. However, it is not yet understood how this technology integration could best perform.

There is extensive literature covering the environmental benefit of CO_2_ polymerisation with epoxides to produce polycarbonates, thereby achieving carbon capture and utilisation (CCU), and contributing towards a mitigation of climate change.^12^, ^13^, ^14^, ^15^, ^16^ However, it is important that newly designed industrial processes to mitigate greenhouse gas emissions do not introduce new hazards.^16^ Therefore, the LCA and techno‐economic methodology becomes particularly useful as the process is holistically analysed across the entire life‐cycle.^15^, ^17^

Other key work on techno‐economic aspects of citrus‐based sustainable product applications include: use of limonene as a solvent for industrial cleaning applications;^18^ use of PLC as a gas separation membrane;^19^ extraction of pectin and flavonoids from citrus waste alongside limonene;^20^ use of limonene for pest control applications and as a platform chemical for advanced polymer production.^21^, ^22^

*Research objectives*. Our study implements a detailed and holistic process design for the novel PLC production pathway. We assess the integration of energy cogeneration (via an AD/CHP plant as opposed to gasification used in previous literature)^8^ and waste resource recovery maximisation (via the PLC production process) by exploring the trade‐offs between environmental sustainability and economic viability via a thorough sensitivity analysis on operating conversion. PLC is also compared with PS to highlight the environmental competitiveness of the biopolymers, which is instrumental in solving some environmental problems faced by the plastic industry as well as building a sustainable circular economy. We also discuss how an industry‐wide substitution of standard plastics with CCU biopolymers can mitigate the escape of atmospheric CO_2_ concentrations away from the planetary boundary. Finally, this environmental contribution is quantified based on our process design.

## 2 Methodology

### 2.1 Process Flowsheet Design Framework

Building on empirical data, a conceptual framework was adopted for the chemical process design to ensure research reproducibility, where the assumptions and decisions made at each process design level are presented in a transparent manner.^23^ With each progressive level, the number of assumptions decreases, thereby increasing the granularity and accuracy of the model. At each level, the economic potential of the PLC process design was evaluated to understand the overall economic and technical feasibility, which is particularly important for novel processes (e. g. PLC requiring significant capital investments thus regarded as high‐risk).

MATLAB R2017b was used to model the kinetic interactions in the epoxidation reaction and estimate the size of the reactor required as a function of conversion. This allowed for more detailed economic modelling of the proposed designs, since installation and operating costs of a reactor may have a large impact on the overall economic potential.

The PLC production process was simulated using Aspen Plus V9, which enabled comprehensive analysis of process dynamics and rigorous optimisation of process parameters such as reflux ratios in the separation columns. Notably, the rigorous modelling research here bridged the research gap highlighted in previous studies on optimisation of the separation sequences due to heating duties and operational costs. Using Aspen Plus allowed for both the design of process units and sensitivity analyses for operation parameter optimisation. Specifically, the number and sizes of different units were modelled accurately, whilst conversion was kept as a degree of freedom for further optimisation.

### 2.2 Life Cycle Assessment

Life cycle assessment (LCA) provides a systematic and rigorous approach to evaluate the holistic environmental impacts of a specific product and its processing procedure over the entire life cycle.^17^ In accordance with international standards ISO 14040 and ISO 14044, this LCA includes four phases: goal and scope definition, life cycle inventory analysis (LCI), life cycle impact assessment (LCIA), and interpretation. This study defines the system boundaries covering the cradle‐to‐gate life cycle of bio‐based PLC and petroleum counterparts, where the “gate‐to‐grave“ life cycle stages (i. e. transportation of product to user, use of product, and disposal or dis‐assembly) were assumed similar, regardless of synthesis route.^15^ This study adopts an attributional LCA (ALCA) approach which accounts for impacts directly related to the system being analysed.

### 2.3 Goal and Scope of LCA

*Goal and scope definition*. This LCA is intended to test the hypothesis that the presented PLC process design offers environmental benefits over the traditional petroleum‐based counterparts. PLC and PS production were compared to quantify the relative environmental impacts of each product life cycle. Sensitivity analyses carried out over various parameters assist in maximising the transparency and reproducibility of the results. This LCA study aims to:

- identify the optimal reactor oxidation conversion from an environmental sustainability perspective;
- compare PLC production with PS production over various environmental impact categories;
- investigate the environmental impacts associated with implementing a waste resource recovery system;
- and identify the environmental hotspots contributing to the impacts occurring over the life cycle of PLC production and highlight the improvement opportunities.

The results of our LCA are expected to provide project developers, academics, researchers and policy decision makers with a deeper insight into the environmental impacts and incentives associated with polymer production from biowaste.

*System definition and functional unit*. The product system boundary was defined to cover the processes and sub‐systems involved in the “cradle‐to‐gate“ PLC production. It is decomposed into three sub‐systems: the cultivation of processing grade oranges, the production of limonene as a co‐product of orange juice production and the production of PLC from limonene. The primary function of the product system is to produce PLC from bio‐based limonene. However, as discussed later in this section, waste resource recovery methods are modelled to capture value from otherwise wasted by‐product streams. This results in a large amount of energy production which can be considered as a secondary function of the process.

The functional unit was defined as “per unit (1 kg) of PLC produced at refinery gate“. As PS has equivalent properties to PLC,^5^ the LCA comparisons of PLC with conventional petroleum‐based polymers was based on the functional unit “per unit (1 kg) of polymer”.

*Allocation procedures*. Allocation procedures were applied to partition the inputs‐outputs, impacts, and upstream environmental interventions at multi‐output sub‐systems (e. g. the allocation of environmental impacts among orange juice and its co‐products limonene and citrus waste). To understand the implications of this procedure on the environmental footprints of the PLC, a sensitivity analysis was performed to assess the robustness of the simulation results to three allocation methods; by economic relationship (accounting for price and quantity) or by physical relationship (i. e. mass and energy). A system expansion approach was adopted where energy recovery from waste occurred (electricity and heat from combustion of biogas derived from anaerobic digestion). It was assumed that the electricity would directly displace an equivalent amount of electrical power generated from the average national grid mixture.

A stoichiometric carbon‐counting approach was used to “track” the biogenic carbon flows from orange cultivation into PLC over the life cycle. This C‐counting approach was applied to determine the carbon sequestered into limonene (from the orange cultivation phase of the life cycle) as well as carbon immobilised into PLC through copolymerisation, and downstream release of carbon during the sequential processes (e. g. anaerobic digestion). The carbon sequestered into PLC therefore represents a negative greenhouse gas emission over the cradle‐to‐gate life cycle, but this carbon may be returned to the environment in various ways depending upon the consequent fate of the PLC.

*Life cycle inventory analyses (LCI)*. The data required, including component flows, utilities and emissions, was obtained from the flowsheet simulation. This was complemented by data from SimaPro databases and orange juice production data from literature.^11^

*Impact categories and impact assessment methods*. A problem oriented (midpoint) approach, ReCiPe Midpoint (H), was applied in our study to perform the life cycle impact assessment (LCIA). This method was used for default analysis, with endpoint methods used to optimise operating conversion. To provide a holistic LCIA view, a wide range of impact categories that cover different environmental aspects have been investigated, ranging from global warming and fossil depletion, to acidification and smog formation. LCA was implemented on the modelling platform SimaPro V8.3.

*Planetary boundaries*. The CCU properties of the PLC process were quantified and the potential of a plastics industry‐wide substitution of standard plastics (Polyolefins, PVC, PS, EPS & PET) with PLC and similar bioplastic pathways was assessed with regards to mitigating the escape of atmospheric CO_2_ concentrations from the planetary boundary.^24^, ^25^

## 3 Process Design Results

### 3.1 Reaction Pathway

**Figure 1:**
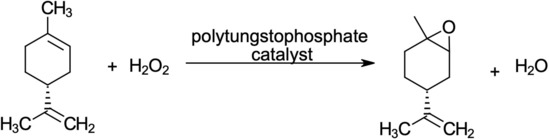
Epoxidation of d‐Limonene by H_2_O_2_ to produce limonene oxide and water.^10^

As shown in Figure 1, the epoxidation reaction (Reaction 1) occurs as d‐limonene is oxidised by H_2_O_2_ in the presence of a polytungstophosphate catalyst (formed from H_2_O_2_, tungstate and phosphate) to produce limonene oxide and water. This solvent‐free reaction, proposed by Resul et al.,^10^ has the potential to improve the environmental and economic performance of the overall process, compared to using TBHP as the oxidising agent. Previous process designs had large environmental burdens due to the TBHP synthesis route and the requirement for an additional solvent. This epoxidation reaction aims to reduce these effects by utilising H_2_O_2_ in the place of TBHP, and excess limonene in the place of an additional solvent, which simultaneously reduces the number of chemicals and reactions involved in the production of PLC, with water being the only by‐product. The conditions for this reaction are 50 °C and 1 bar. Assuming 100 % conversion and selectivity for this reaction allowed for side reactions, such as the hydrolysis of the epoxide and formation of diols, to be neglected. This facilitated streamlined process design to provide input/outputs for the follow‐up LCA.

**Figure 2:**
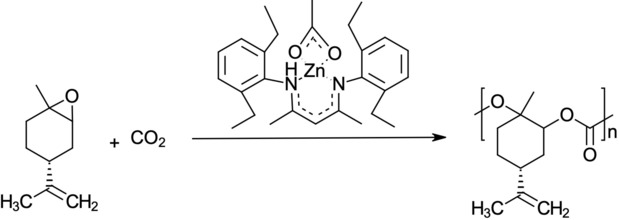
Copolymerisation of limonene oxide and CO_2_, in the presence of a β‐diiminate zinc acetate complex catalyst,^[4]^ to produce PLC.^6^

Figure 2 demonstrates the copolymerisation reaction (Reaction 2) between limonene oxide and CO_2_ to produce PLC. This is the original reaction, proposed by Byrne et al., that uses the Zinc complex catalyst.^4^ Under the proposed conditions of 25 °C and 7 bar, this reaction is able to achieve 50 % conversion after a residence time of 7 hours.

### 3.2 Inputs and Design Specifications

The production rate of PLC was assumed to be 21,600 tonnes/yr,^8^ which is less than 10 % of US production capacity and less than 1 % of global PS supply,^26^ meaning that the problem of over‐supply is avoided. This process takes into account the scaling‐up effects which enabled a robust LCA evaluation based on the functional unit of 1 kg PLC produced. Since PLC has similar properties to PS, it was assumed that PLC will replace a fraction of the PS demand and that the two polymers are likely to be priced similarly. Based on the price of general purpose PS (GPPS) ranging between 1400 USD/tonne and 1700 USD/tonne, it was estimated that the average selling price for PLC is approximately 1550 USD/tonne at a purity of >99 % which agrees with previous estimates.^7^

Limonene was assumed to be available at a purity of 100 % and a price of 76 USD/tonne. This price was determined by estimating the variable costs for the process of extraction of limonene from citrus waste as outlined in previous work.^7^, ^27^ Based on the consideration of limonene mass flows required by the PLC process, São Paulo, Brazil was chosen as the location of the PLC plant. This state is the largest producer of citrus‐derived products globally, with a capacity of approximately 19.2 million tonnes of fresh citrus fruit in 2017.^28^ For the proposed target production capacity, 7.3 million tonnes of oranges require processing, which is sustained by this choice of location.

H_2_O_2_ is available as an aqueous solution at 50 wt % purity and a price of 520 USD/tonne. H_2_O_2_ becomes extremely explosive and promotes combustion at high temperatures as it rapidly decomposes to water and oxygen. Therefore, any pipes or process equipment that handle H_2_O_2_ should be kept at temperatures below 100 °C. As it is corrosive to metal surfaces, its presence in the equipment should be minimised to reduce potential increased maintenance costs. Additionally, corrosion‐resistant stainless steel was assumed as the building material for process units.

CO_2_ is available at 100 % purity and 30 USD/tonne, assuming the PLC production site would be situated adjacent to industrial plants that dispose of their CO_2_. Therefore, only the compressing and installation costs of a gas infrastructure were accounted for.

Methanol is required for precipitation of PLC out of solution after the polymerisation reaction. It was assumed that methanol is available at 100 % purity and 400 USD/tonne.

### 3.3 Level 1: Mode of Operation

Level 1 of the design framework considers whether the process is operated continuously or in batch mode. By considering the large production rate, non‐seasonal market forces, and process practicality, a continuous process to produce PLC was designed. Detailed factors can be found in Supplementary Information (SI‐1).

### 3.4 Level 2: Input‐output Structure

Level 2 of the design framework requires specification of the process output stream destinations and recycles, thereby defining the input‐output structure. Key Level 2 decisions are detailed in Supplementary Information (SI‐2) and the resulting input‐output structure is shown in Table 1.

**Table 1:** Component destinations and boiling points at 1 bar.

Component	B.P./°C	Destination
CO_2_	−79	Gas recycle
Water	100	Non‐valuable by‐product
H_2_O_2_	150	Liquid recycle
Limonene	176	Liquid recycle
Limonene oxide	189	Reaction 2 feed
PLC	–	Primary product

### 3.5 Level 3: Reactor Modelling and Recycle Structure

Level 3 of the design framework integrates detailed specification of the reactor systems into the overall flowsheeting process. Key decisions at this level are discussed in this section.

*Reactor systems*. Reactions 1 and 2 require different operating conditions thereby requiring at least 2 reactors in the process. A plug‐flow reactor (PFR) was chosen for Reaction 1 since this type of reactor yields a better conversion per unit volume compared to a continuous stirred‐tank reactor (CSTR). Additionally, the products of Reaction 1 are unlikely to cause fouling in a PFR and Douglas heuristics suggest using a PFR for isothermal operation.

Reaction 2 is a multiphase reaction, so will likely take place in a semi‐flow batch reactor in practice. However, modelling such a reactor requires some unavailable kinetic information. The polymerisation reactor was therefore modelled as a CSTR with a specified residence time and conditions that are known to be optimal from experimental studies.^4^ Stainless steel was chosen as the building material for both reactors due to the presence of highly corrosive H_2_O_2_ in the system.

**Figure 3:**
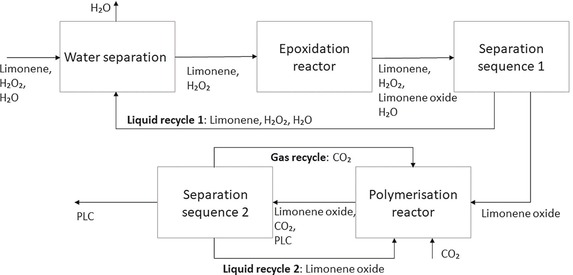
Recycle structure at Level 3 of the design framework.

*Recycle streams*. In total there are 3 recycle streams in the process (Figure 3). Separation of water after the first reaction system was considered; however, it was decided that recycling water back to the water separation unit placed before the reactor would reduce the overall number of separation units required in the future levels of design. There is one gas recycle which necessitates a compressor in the system and which needs to be accounted for in the economic analysis.

**Figure 4:**
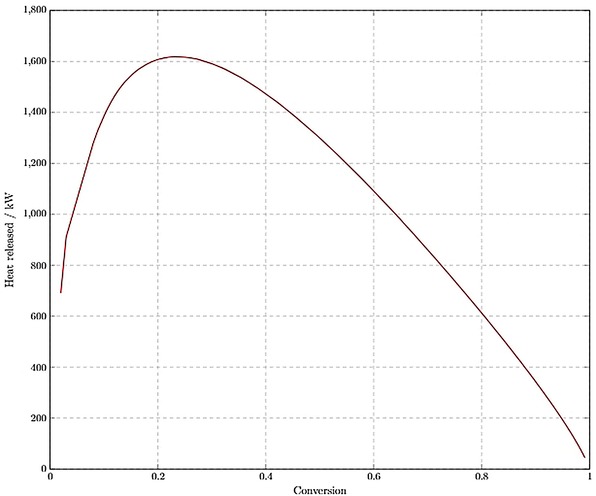
Heat released in R1 as a function of operating conversion.

*Reactor thermal effects*. Reactions 1 and 2 are exothermic, requiring cooling systems to maintain isothermal operation. Hess’ Law was used to calculate the enthalpies of Reaction 1 and 2 as −180 kJ/mol and −110 kJ/mol, respectively. The epoxidation reactor (R1) was modelled in MATLAB R2017b using the kinetic data derived from original empirical research,^10^ and the heat released was analysed against the operating conversion. Figure 4 shows that by increasing the conversion, the heat released increases to a maximum of approximately 1600 kW at a conversion close to 0.2, then tends to zero as the conversion approaches 1. This trend arises since the heat released in R1 is a function of reactor volume and reaction rate. Reactor volume increases with conversion as a higher residence time is required. The reaction rate decreases with increasing conversion since the load flowing through the reactor decreases. These opposing effects constructively create the relationship shown in Figure 4. It was found that to meet the target PLC production, the polymerisation reactor (R2) releases approximately 420 kW of heat.

*Reactor and compressor costing*. Installation costs of the reactors were calculated using Guthrie's correlations^23^ based on volumes obtained from the MATLAB model (in Supplementary Information (SI‐7)). These costs were minimised by choosing an aspect ratio of 6 according to Douglas heuristics. The model flexibility allowed the installation costs to be calculated at different operating conversions, ensuring optimality.

The cost of the compressor required to pressurise the CO_2_ feed to 7 bar was estimated using Guthrie's correlations which consider equipment inefficiencies and gases present.

*Level 3 economic potential*. The result of Level 3 is a process economic potential that considers the costs of reactors and compressors. Since it was possible to obtain these costs as a function of the operating conversion in R1, an optimum conversion could be chosen that maximised the process economic potential.

**Figure 5:**
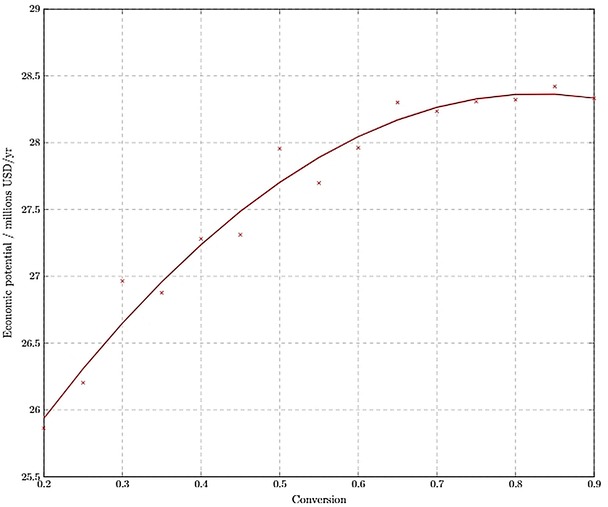
Level 3 economic potential as a function of operating conversion in R1.

At this stage of the design framework the theoretical optimum conversion is approximately 0.85 (Figure 5), suggesting higher operating conversions are favoured. This optimality exists because as the conversion tends to 1, the size of the reactor becomes very large; to prevent computational errors, the analysis was performed for the conversions range of 0.2‐0.9. At a conversion of 0.85, the economic potential of the process is about 28.4 M USD/yr.

### 3.6 Level 4: Separation System

The PLC production flowsheet was modelled in Aspen Plus V9 to rigorously model the recoveries of components in separation systems (separations are no longer assumed perfect at this Level). The separation sequences were designed using Douglas heuristics.

*Separation method and decision analysis*. The best separation method for different component pairs was determined using Jaksland et al. analysis (see Supplementary Information (SI‐10a) for results).^29^ There are a number of assumptions and limitations introduced here: first, we assumed that PLC precipitation by methanol achieved 100 % recovery; second, although the analysis showed that liquid membranes and crystallisation were both viable separation techniques, these methods were not considered due to lack of experimental data and modelling constraints; third, the costs and energy requirements associated with each separation technique are not considered by this analysis framework.

*Separation sequencing*. The PLC process has two different reactive systems with corresponding separation sequences downstream. The limonene oxide produced by R1 must be purified before it is fed to R2, to prevent a build‐up of water, H_2_O_2_ or limonene which are inert in the second reactive system. Similarly, PLC must be purified after R2 to meet product specifications. The two separation sequences were considered separately so that a pure product is achieved at the end of each sequence. The Douglas heuristics adhered to for each sequence are detailed in Supplementary Information (SI‐10b).

*Flowsheet modelling*. The process was modelled in Aspen Plus V9 to obtain operating conditions for separation units. The Non‐Random‐Two‐Liquid (NRTL) thermodynamic property method was used throughout the process model for numerous reasons: the non‐ideal components present throughout the system are modelled effectively by using liquid activity coefficients to calculate phase equilibria; multiphase systems can be modelled to a good standard; and the dissolution of CO_2_ in methanol can be accounted for, which is important for modelling separation sequence 2.

**Figure 6:**
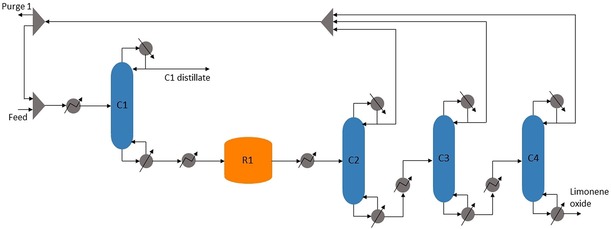
Process flow diagram for limonene oxide production and purification. C1, water separation; R1, epoxidation reactor; C2, H_2_O_2_ and water separation; C3 and C4, limonene separation from limonene oxide.

**Figure 7:**
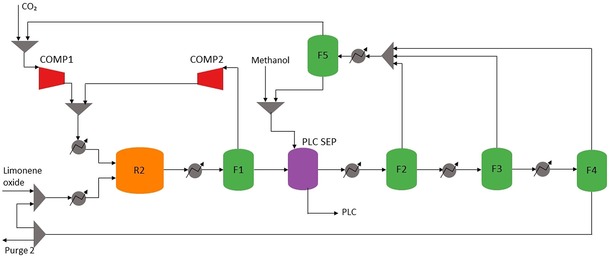
Process flow diagram for PLC production and purification from limonene oxide and CO_2_. R2, polymerisation reactor; F1, liquid‐gas phase splitter; PLC SEP, methanol precipitation of PLC; F2 and F3 and F4, CO_2_ and methanol separation; F5, CO_2_‐methanol splitter.

*Separation strategy*. Figures 6 and 7 show the overall process as modelled in Aspen Plus (see Supplementary Information (SI‐3) for the Aspen flowsheet). The determined overall separation strategy is detailed in the following.

*Water separation (C1)*. Distillation column C1 removes water from H_2_O_2_ and limonene, thereby minimising R1 size and heat duty. Since H_2_O_2_ cannot be heated above 373 K due to its explosive properties, this column is operated at 0.1 bar to reduce the boiling point of water. Water is extracted at the distillate to be used elsewhere in the process, thereby minimising waste post‐treatment. The pure reaction mixture of limonene and H_2_O_2_ is the bottom product of C1 and is subsequently fed to R1.

*H_2_O_2_ and water separation (C2)*. Distillation column C2 separates the light reactor effluent components (H_2_O_2_ and water) from the heavy reactor effluent components (limonene and limonene oxide). This column is also operated under vacuum conditions due to the presence of H_2_O_2_. The distillate is recycled back to C1 so water can be separated and pure reactants fed to R1.

*Limonene separation (C3 and C4)*. Distillation columns C3 and C4 separate limonene from limonene oxide so that pure limonene oxide can be fed into the second stage of the process. This is a difficult separation step due to the small difference in the boiling points of the two components (only 13 K). Two columns are therefore required to achieve a limonene oxide purity of >90 mol%, ensuring that a minimal amount of limonene enters the second reactive system and preventing it from building up in the R2 liquid recycle. Since the components of this separation have high thermal stability, the columns operate at pressures closer to atmospheric (0.3 bar and 1 bar respectively), reducing operating and capital costs. We found that increasing the pressure across the two columns ensured a higher recovery of limonene oxide in the bottom product of C4. The distillate, mostly limonene with some limonene oxide impurity (about 10 mol%), is recycled back to C1 to be used as solvent and reaction mixture.

*Phase splitter (F1)*. Since the effluent of R2 is a two‐phase mixture, a flash vessel (F1) can easily separate the vapour phase from the liquid phase without the need for expensive distillation. The vapour phase is near pure CO_2_ and can be recycled directly back to R2, whereas the liquid phase (a mixture of unreacted limonene oxide, PLC and some CO_2_ and methanol dissolved in limonene) requires further processing. F1 is operated at 7 bar to reduce the energy required to re‐pressurise the CO_2_ recycle stream.

*PLC separation (PLC SEP)*. PLC is precipitated from the R2 effluent using an excess of methanol (3 : 1 molar ratio). It is assumed that the PLC completely precipitates into an easily filtered solid phase.

*CO_2_ and methanol separation (F2, F3 and F4)*. Flash vessels F2, F3 and F4 separate methanol and any dissolved CO_2_ from the limonene and limonene oxide mixture, so that these components can be recycled back to the process. Three flash vessels were determined more efficient than a distillation column since the distillation column only required two stages. The temperature is increased over the three vessels, while the pressure is decreased. This decreased the heating duty required and improved the recovery of components in their respective phases.

*CO_2_ and methanol splitter (F5)*. Flash vessel F5 separates any dissolved CO_2_ from methanol in the gas recycle stream. It is operated at room temperature and pressure since CO_2_ is not very soluble in methanol at these conditions, thereby reducing the operating cost of this vessel. The methanol is recycled to PLC SEP whilst the CO_2_ gas is re‐pressurised and fed into R2.

*Waste resource recovery considerations*. The PLC process was designed such that the amount of waste produced was minimised and any waste streams were analysed to ensure that maximum utilisation was achieved. A waste resource recovery system (WRRS) was modelled to process waste in a sustainable way, where the energy produced either offset the process utility requirements or was modelled as substituting a fraction of the national grid energy. There are three waste streams from the PLC process, detailed in the following.

*C1 distillate*. This is the largest waste stream in the proposed PLC plant design, consisting of mostly water (>60 wt %) and limonene (about 30 wt %). Limonene should not be emitted to the environment: it is toxic to aquatic organisms and bioaccumulation can occur in fish; it has a low flashpoint of 48 °C and so should not be exposed to atmosphere until it is cooled down. In the WRRS, limonene is exposed to anaerobic conditions at about 40 °C, hence the explosion hazard is minimised. The WRRS was modelled to convert any unreacted limonene to biogas, that can be used to produce heat and electricity to be used elsewhere in the plant. Due to the high reactant purity requirements of the reaction systems, recycle of the limonene/limonene oxide was not considered.

*Purge 1*. This purge, required to prevent the build‐up of limonene oxide in the system, has a very high content of limonene (about 70 wt %) with some limonene oxide and water (about 15 wt % each). Similar to the C1 distillate, this stream is fed to the WRRS to convert the organic compounds into biogas.

*Purge 2*. This stream, necessary to prevent the build‐up of limonene and methanol in the polymerisation reaction cycle, is mostly limonene (about 50 wt %) and limonene oxide (about 45 wt %) with a small fraction of methanol impurity (about 5 wt %). This purge is also fed to the WRRS to generate biogas.

*Level 4 economic potential*. The detailed process model enabled most of the process equipment, including heat exchangers and distillation columns, to be sized and costed. Mass balances around process units (using Aspen Plus) also allowed for waste stream flows and compositions to be analysed. The conversion of R1 was kept as a degree of freedom, to ensure that the most economically viable conversion was selected for the design.

Separation and heat exchanger costs were calculated using Guthrie's correlations to relate the sizes of equipment to approximate installation costs, whilst the variable costs were estimated from the heat duties that were extracted from the Aspen Plus process model. The disposal costs account for the annualised capital cost of the WRRS, based on the mass flow into the system. Any excess energy produced by the WRRS is assumed to be sold to the local grid or to other industrial sites.

It was found that the proposed WRRS system is likely to have a large capital cost associated with it. This is driven by the significant amount of citrus waste (CW, 168 kg) processed per 1 kg of limonene feed that the process requires. Three scenarios were explored in the economic analysis:

1. No waste resource recovery implemented (Scenario 1)
2. Full waste resource recovery implemented (Scenario 2)
3. Partial waste resource recovery implemented (5 % of CW) (Scenario 3)

**Figure 8:**
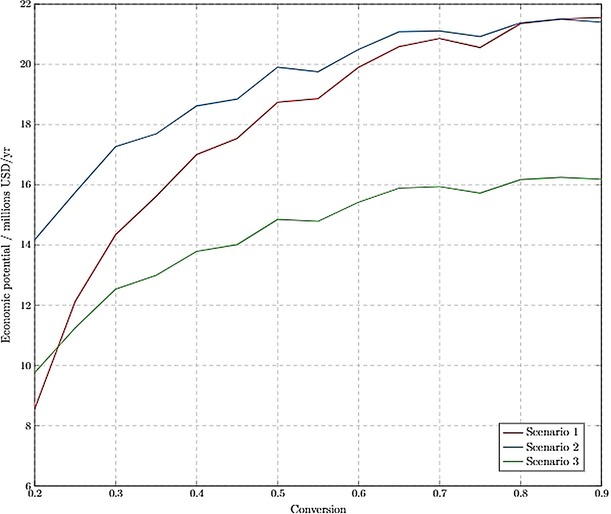
Level 4 economic potential as a function of operating conversion in R1.

All scenarios assumed that limonene was initially extracted from CW and only differ in the fraction of waste processed. It was found that processing 5 % of the total CW would produce sufficient utilities to supply the whole PLC process energy demands, so this was chosen as one of the potential scenarios. Figure 8 shows that all the scenarios had positive economic potential and that the optimum conversion of limonene was 0.8 at this level of the design framework.

### 3.7 Level 5: Energy Integration

The energy requirement of the plant was minimised by optimising the use of existing hot and cold streams by implementing heat exchangers. All heat exchangers were assumed to be adiabatic and a minimal driving force for heat exchange was enforced at 10 K. The following analysis was performed at the optimal conversion (0.8).

**Figure 9:**
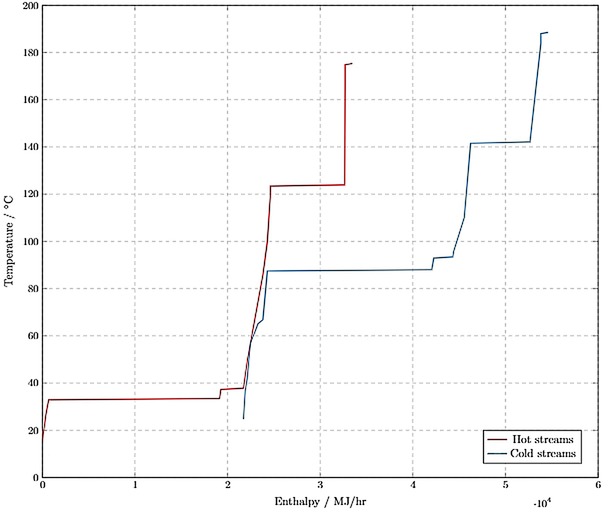
Composite curves used for pinch analysis.

*Composite curve analysis*. The total energy saving potential was found to be 11,700 MJ/hr by implementing a composite curve analysis. The pinch was found to be at 57 °C as shown by Figure 9. The temperature of the hot streams was adjusted by 10 K to ensure that the minimum driving force requirement was fulfilled and The Second Law of Thermodynamics obeyed. It was assumed that the energy savings fraction was the same for all R1 operating conversions, thereby streamlining the sensitivity analysis.

**Figure 10:**
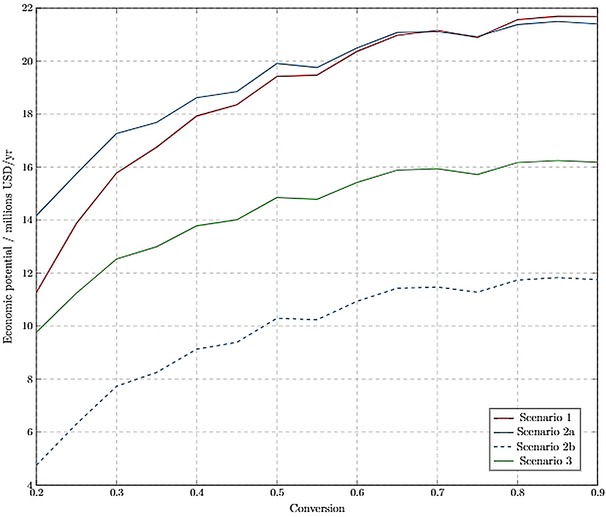
Level 5 economic potential as a function of operating conversion in R1.

*Level 5 economic potential*. The heat integration minimised the energy requirement of the process, thereby reducing the operating costs of distillation columns and heat exchangers and increasing the overall economic potential (Figure 10).

Heat integration had the largest effect on Scenario 1, since a large fraction of costs in this scenario were attributed to utility costs of distillation columns. This effect is not visible on scenarios with WRRS, since those scenarios were able to get all the utilities required, as a result of energy cogeneration.

Scenario 2a, where all the citrus waste is processed into fuel, had nearly equal economic potential to Scenario 1, where there is no WRRS. It was assumed that any excess utilities produced by the WRRS could be sold to the grid or nearby industrial plants, hence these energy sales account for 23 % of the revenue in Scenario 2a. However, selling excess utilities may not be possible in all potential plant locations, therefore the scenario was tested against an assumption of no energy being sold (Scenario 2b). This scenario then became the least lucrative; the large waste resource recovery system had a large capital cost associated with it, therefore the national policy for selling energy to the local grid should be taken into consideration during location planning for plant design.

### 3.8 Economic Sensitivity Analysis

The three design scenarios proposed were tested against changes in key economic variables, to assess their robustness. The analysis was conducted using the optimal conversion of 0.8.

The design with no WRRS (Scenario 1) was the most economically robust scenario whilst the design with partial resource recovery (Scenario 3) was the most sensitive (Table 2). Scenario 2 performed similar to Scenario 1, assuming that all excess utilities could be sold at market price. If utilities could not be sold, Scenario 2 was the least lucrative due to the large capital costs associated with implementing the WRRS (Figure 10). Additionally, Scenario 2 was sensitive to utility prices, since the revenues from utility sales account for approximately 40 % of the positive economic potential. This means that the focus of the plant may shift from PLC production to energy generation in the case it becomes more economical due to fluctuations in utility prices. Scenario 2 has a safety margin of nearly 10 M USD/yr from utility sales, meaning that the risks of the plant are diversified. It is also possible to re‐focus the plant towards energy generation in the case that PLC does not develop expected demand or market price.

**Table 2:** Deviations in component prices required to reduce economic potential to zero.

Scenario	PLC sales price	Limonene extraction costs
1	−66 %	+1380 %
2	−65 %	+1370 %
3	−50 %	+1040 %

Figure 10 shows that all three proposed design scenarios are sensitive to the conversion in R1. The economic potential for each design nearly doubles over the range of conversions modelled. This is due to larger utility and raw material costs at lower conversions, that arise from larger mass flows through the process. The designs that incorporate WRRS are less sensitive to the conversion changes than Scenario 1, since they are able to supply their own utilities and hence do not suffer from increased utility costs at lower conversions. However, they are still sensitive to conversion, since at lower conversions more raw materials are needed. It was found that the designs were not sensitive to the price of H_2_O_2_ or CO_2_ since these feeds were relatively small (70 wt % of all feeds are limonene). At lower conversions, the designs would become more sensitive to these feed prices, since raw materials become a sizable fraction of the costs. It should be noted that due to the low amounts of polytungstophosphate required (1:0.005 molar ratio H_2_O_2_ to catalyst),^10^ the costs of catalyst separation and regeneration have been assumed negligible in this analysis, however future work should consider this economic impact due to possible large purchase or synthesis costs.

The cost of extracting limonene from citrus waste was estimated as 76 USD/tonne using the variable costs of the extraction process. This involves steam extraction of orange oil from the peels, followed by further purification by vacuum distillation or winterisation.^7^ It was therefore important to measure the sensitivity of the proposed designs towards the uncertainty and potential deviations in limonene extractions costs. It was found that the designs are generally not very sensitive to this variable, with Scenario 1 and Scenario 2 being the least sensitive and Scenario 3 the most. This is because a large fraction of the costs of the designs are due to installation costs of process units. For example, in Scenario 2, 81 % of all costs were equipment related and hence small changes in costs of raw materials do not have significant impacts on the overall economic potential.

The price of PLC was assumed to be similar to the price of GPPS since the actual bulk price of PLC is unknown. Whilst it is likely that the actual price of PLC will be higher than that of GPPS (this analysis provides a lower bound of economic potential, relative to the price) due to its environmental benefits, it is still important to know what price PLC should be for the design to be profitable. Scenario 3 is the most sensitive to PLC price since it does not rely on revenue from excess utility sales, whilst also sustaining high capital costs from the implementation of a partial WRRS.

It should be noted that all three proposed designs rely on a relatively low‐cost supply of limonene ‐ this is justified since the limonene is sourced from what is currently regarded as a waste which requires a remediation pathway. Purchase of limonene at its current market price of 3500 to 4000 USD/tonne would produce a strongly negative economic potential in all the proposed designs. The price of PLC must rise to 3875 USD/tonne for any of the scenarios to be positive in this situation, more than double the current average price of PS. Therefore, a more detailed process design for limonene extraction can be explored in future to test the validity of calculated economic potentials and the final design recommendations.

## 4 Life Cycle Analysis Results

### 4.1 Life Cycle Inventory Analysis

*Limonene production*. This study considers mass, economic and energy allocations to assess the sensitivity of the LCA to these different methods. An orange juice processing plant was analysed and modelled within SimaPro,^11^ then allocation methods were used to assign the environmental burdens among the different outputs of this process. A summary of the allocation factors is presented in Table 3, whilst the inputs and outputs from the orange juice process are outlined in Table 4 (see Supplementary Information (SI‐13 & SI‐14) for details on allocation factor calculations and orange juice process input data).

**Table 3:** Different allocation methods to assign the appropriate environmental burdens to limonene.

Output	Mass allocation/%	Economic allocation/%	Energy allocation/%
Limonene	0.3	3.0	7.8
Orange juice	47.7	85.0	52.5
Citrus waste	52.0	12.0	39.7

**Table 4:** Inputs/outputs from orange juice process plant analysed.^11^

Outputs from orange juice plant (per kg of limonene)		
Limonene	kg	1.0
Orange juice	kg	153.6
Citrus waste	kg	168.0

Inputs to orange juice plant (per kg of limonene)		
Oranges	kg	335.7
Electricity	kWh	22.0
Natural gas	MJ	99.7
Water	m^3^	0.75
Detergents (soda)	kg	1.30
Detergents (nitric acid)	kg	0.04

*PLC production*. Regression models derived from the Aspen simulations were fed into the LCA, enabling the flowsheet to be optimised from an environmental perspective. Regression models were derived by initialising the Aspen simulations with varying R1 operating conversions where the material and energy flows were expressed as functions of the conversion rate (please see Supplementary Information (SI‐15)).

*Carbon capture and utilisation*. One of the main advantages of switching to a polymer derived from biomass is the CO_2_ that is sequestered in the process. Oranges store atmospheric CO_2_ as carbon via photosynthesis, from which the PLC is then synthesised. Since this CO_2_, coupled with the CO_2_ used as feedstock in copolymerisation, are captured and fixed into PLC, the environmental saving of carbon sequestration should be credited to the PLC life cycle. Based on the stoichiometric carbon‐counting approach, 9.05 kg CO_2_ equivalent is sequestered per kg PLC. Following the PLC refinery design specification, the annual PLC production of 21,600 tonnes has the potential to impact climate change mitigation, with a significant amount (196 ktonnes) of CO_2_ sequestered.

**Figure 11:**
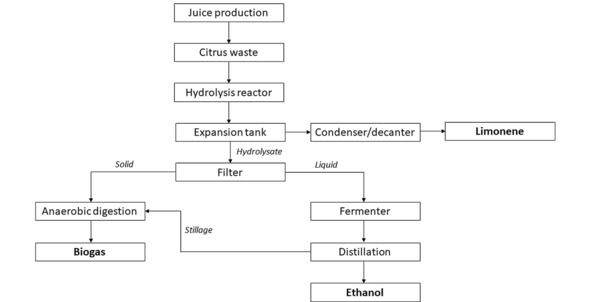
Integrated WRRS as presented by Rezzadori et al.^30^

*Citrus waste resource recovery*. To make full use of the large amount of citrus waste produced from the orange juice plant, a waste resource recovery system was modelled, as shown in Figure 11.^30^ Using this integrated process maximised utilisation of the citrus waste, producing 39.6 L of ethanol, 45 m^3^ of pure methane and 8.9 L of limonene per tonne of wet citrus waste.^30^

The biogas and ethanol produced by this process can be used as a green source of fuel to operate the rest of the PLC production system, whereas the extra limonene extracted can be fed to the PLC process. Implementing this WRRS therefore causes the mass allocation for limonene to increase to 0.7 % (compared to the original 0.3 %). This is still physically viable since the mass content of limonene within oranges has been shown to be between 0.3 and 1 %.^31^

*PLC processing waste resource recovery*. The PLC waste was modelled as being treated by anaerobic digestion (AD) followed by a combined heat and power (CHP) plant. The composition of this waste depends on the conversion within the limonene oxide reactor but is made up of primarily limonene and water, with smaller amounts of limonene oxide, methanol and hydrogen peroxide.

In this study, all biodegradable compounds were assumed to be digested using AD, thus theoretical biogas production was used to estimate the maximum energy recovery. The biogas produced (assumed as 65 % CH_4_ (v/v); 35 % CO_2_ (v/v)) from the AD system was estimated based on the theoretical chemical oxygen demand (COD). For the formula C_n_H_a_O_b_N_c_ (1 mol), the theoretical COD is determined by:

$$COD=\frac{2n+(a-3c)/2-b}{2}\times 32g$$

Each mole of CH_4_ consumes two moles of O_2_; thus 1 g COD destruction is equivalent to 0.395 L CH_4_ at 35 °C and one atmosphere.^32^ The biogas is modelled as the feedstock to the CHP plant where both electricity and heat are produced. The electrical power produced is based on the average 1.2 kWh per m^3^ biogas, assumed to be 30 % of the net calorific value for biogas, with 50 % generating thermal energy and the remaining 20 % of the heat being wasted due to inefficiencies, to the environment (examples in Supplementary Information (SI‐16)).^33^ The surplus electrical energy was modelled as substituting the national grid energy mix whilst the surplus thermal energy was assumed to be wasted, after accounting for the process heating requirements and assuming that the cogeneration plant is in close proximity to the PLC production process. This heat was originally assumed to be generated from natural gas combustion within an industrial furnace; it is therefore environmentally beneficial to replace this fossil derived gas with biomethane produced from biowaste.

### 4.2 Life Cycle Impact Assessment

**Figure 12:**
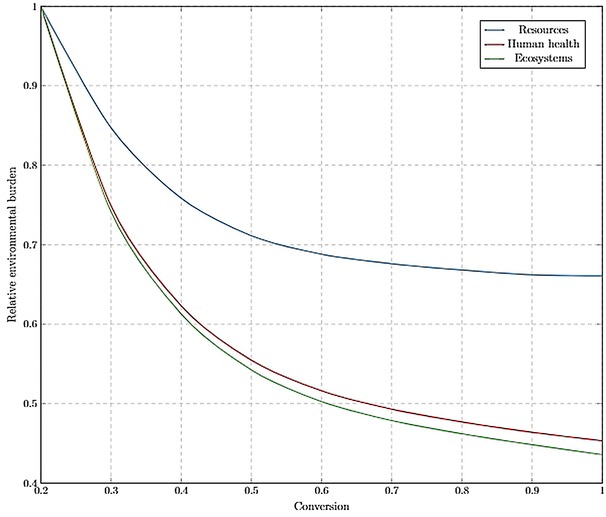
Optimising conversion with respect to endpoint environmental impact categories, without the WRRS implemented. A relative environmental burden of 1 represents the most environmentally damaging conversion.

**Figure 13:**
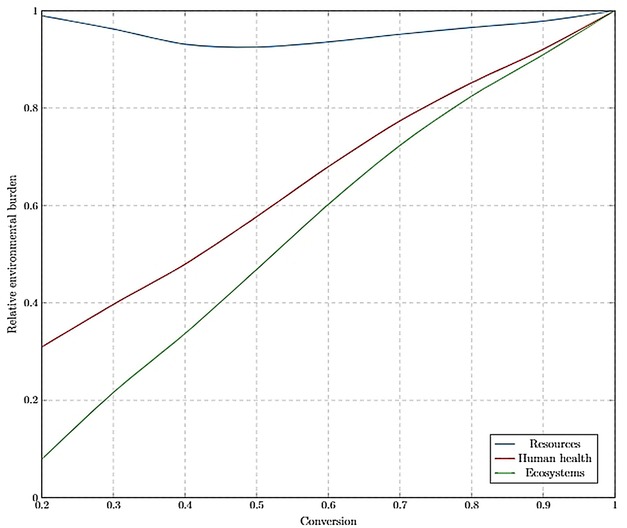
Optimising conversion with respect to endpoint environmental impact categories, with integrated WRRS. A relative environmental burden of 1 represents the most environmentally damaging conversion, whilst −1 represents a positive environmental impact.

**Figure 14:**
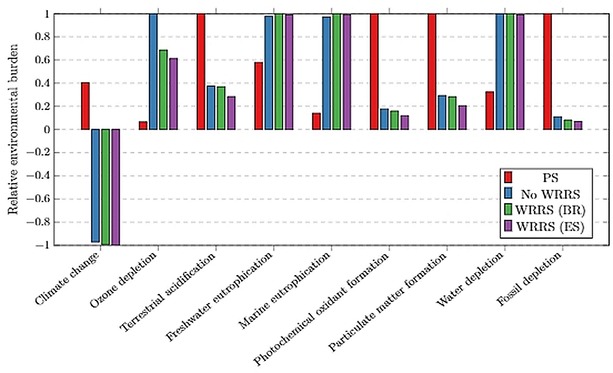
ReCiPe Midpoint analysis of the designed PLC production process, per kg polymer produced. PS production is shown for comparison and three cases the PLC process are provided: with no WRRS; with WRRS in Brazil; and with WRRS in Spain. Scores 0 to 1 represent environmental burdens whilst bars below 0 represent an environmental saving effect.

**Figure 15:**
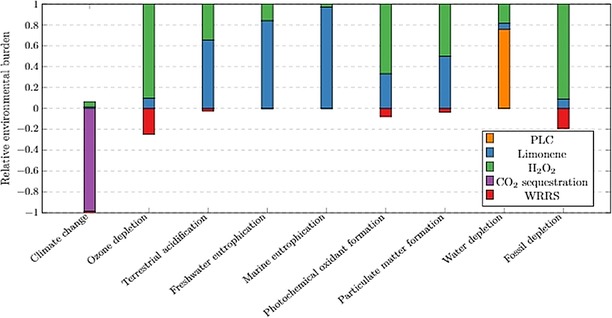
Environmental hotspot analysis, using ReCiPe Midpoint, of the designed cradle‐to‐gate PLC production process, normalised to 100 % and per kg PLC produced. Scores 0 to 1 represent negative environmental burdens whilst bars below 0 represent a positive environmental impact.

**Figure 16:**
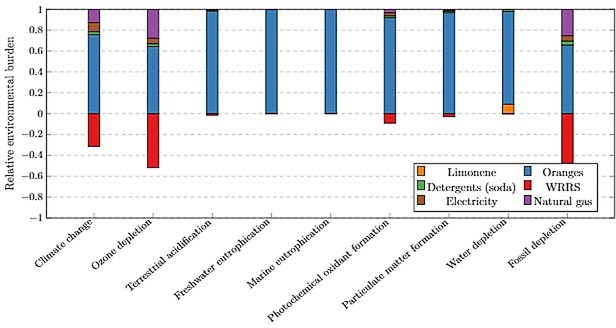
Environmental hotspot analysis, with ReCiPe Midpoint, of the analysed orange juice production process, normalised to 100 % and per kg PLC produced. Scores 0 to 1 represent negative environmental burdens whilst bars below 0 represent a positive environmental impact.

The following sections present and discuss the results of various scenarios modelled in this study along with the LCIA for conversion configurations (Figures 12 and 13) and normalised comparison (%) presented in Figures 14, 15 and 16. The LCIA scores for each individual impact category are given in the Supplementary Information (SI‐17).

*Optimum operating conversion*. Analysis was carried out to determine the optimum operating conversion in R1 from an environmental sustainability perspective, by varying the conversion from 0.2 to 1. The LCIA was performed based on the ReCiPe Endpoint (H) method to normalise and group the impact categories as 3 weighted environmental scores, facilitating comparison to be made across the range of conversions. The outcomes of the assessment were plotted relative to the greatest environmental impact. Across 3 impact categories (human health, ecosystems and resources), environmental burdens significantly reduced with the increasing conversion (Figure 12). This trend agrees with the economic analysis carried out previously in this study, suggesting that from both an environmental and economic perspective, operation at the maximum achievable conversion lead to the optimal performances. This trend is expected since, as conversion is increased, the amount of waste, emissions to atmosphere and energy intensity of separations are reduced.

- *Resources*. As conversion is increased, the dependency on raw materials and resources is reduced. The dependency on these potentially environmental‐damaging resources underpins the performance of the process with respect to these three categories. This is especially true as the only product of the process in this scenario is the PLC.
- *Human health*. As the conversion is increased, less limonene is required to produce a given amount of limonene oxide. This means that less detergents and other harmful substances need to be incorporated into the limonene extraction process. There will also be a decreased amount of hazardous chemicals (such as H_2_O_2_) purged from the recycle stream.
- *Ecosystems*. Similar to the human health impacts, ecosystems are less affected as conversion is increased. Lower waste stream loads emitted to land, water and air, as well as decreased requirements for harmful substances upstream, results in lower environmental burden on ecosystems.

*Conversion optimisation with WRRS*. Figure 13 shows that after implementing the WRRS, the optimum operating conversion has shifted from 1, across all 3 damage categories. Damage to resources is minimised at intermediate conversions (about 0.5), whilst the human health and ecosystems environmental burdens are minimised at lower conversions (0.2). The underlying factor determining these three observations is that the national energy mix is being substituted with the PLC process’ energy cogeneration from biowaste. It should be noted that this utilisation of biowaste as an energy source is not superior to plastic recovery from an economic value‐added point of view and so is only considered as a post‐processing option.

- *Resources*. At lower conversions, increased amount of waste is processed by the WRRS, allowing for higher substitution of the non‐renewable energy sources (natural gas, oil and hard coal) from the national energy mix than if less waste is produced at higher conversions. With the reduction in conversion rate, optimal process operation shifted from biopolymer production to energy cogeneration from biomass. However, from a resources endpoint perspective, different resources are still being depleted leading to an environmental trade‐off, depicted by the U‐shape trend.
- *Human health*. For the human health damage category, the trend exhibited in Figure 13 is caused as more energy from biomass is available to substitute primarily hydrolytic energy sources. Hydro power, although renewable and favourable in the resources damage impact category, is not as preferable as energy from biomass in terms of its effects on human populations. Reservoirs for hydro power flood previously dry land which negatively impacts farming and local communities as well as the habitats of plants and animals.^34^ By reducing the reliance on hydro power, at lower conversions, the PLC process therefore exhibits minimum burdens in the human health impact category.
- *Ecosystems*. Higher ecosystems damage can be induced by hydro power sources, than energy derived from biomass. The construction of large dams can block salmon migration routes and create slow moving warmer reservoirs that reduce water quality and negatively impact the native aquatic life. As demonstrated in Figure 13, producing energy from the PLC process’ biowaste is a more attractive route when considering ecosystems.

*LCIA midpoint approach*. In this section, the LCIA profiles based on a problem‐oriented approach for the designed process are considered. This provides a more comprehensive breakdown of the different environmental burdens associated with the PLC process and a more detailed comparison with PS production. It should be noted that statistical uncertainties associated with endpoint methods are higher due to the propagation of assumptions and errors that occur further along the cause‐effect chain. Midpoint analysis therefore not only allows for a more detailed assessment but also a less subjective analysis.

Considering a midpoint analysis of the PLC production life cycle, Figure 14 shows a comparison of PLC to PS and the implications of including a WRRS. A positive value on the y‐axis represents an environmental burden whereas a negative value represents an environmental benefit. PLC outperformed PS across various impact categories including climate change, acidification, smog formation and fossil depletion. However, PLC delivered less environmentally favourable profiles over PS in the ozone depletion, eutrophication and water depletion impact categories. The decisions therefore depend on the environmental sustainability matrix under investigation.

Implementing a WRRS to the process improved environmental performance in certain impact categories by up to 31 %. The main impact of implementing the WRRS to the PLC process is the energy cogeneration from biowaste replacing the specific country's grid energy mix. This results in less fossil fuel being extracted/processed, and thus the environmental burdens associated with these resources are reduced.

To highlight the importance of geographical location, a scenario was implemented in which the process replaces the specific grid mix of Spain instead of Brazil (Figure 14). 77 % of Brazil's energy production comes from renewable sources (primarily hydro power) with oil, gas and coal making up the remaining 23 %.^35^ Due to the renewable nature of the Brazilian energy mix, the environmental benefits of the WRRS energy production are reduced considerably. For comparison, the Spanish grid energy is simulated as being replaced, resulting in a much greater environmental benefit being associated with PLC, compared to PS. This shows that the LCIA results of PLC are sensitive to process location and thus highlights the importance of geographical placement during process design. However, the LCIA conclusions of PLC versus PS are not sensitive to the PLC scenarios (WRRS and location).

PLC demonstrates environmental benefits on climate change because of the CCU involved in the process. Whilst PS production mines fossil resources and releases CO_2_ in the process, PLC production results in a net depletion in atmospheric CO_2_. CO_2_ is removed from the atmosphere via photosynthesis by the orange trees and is ultimately stored in PLC. The environmental saving effect of carbon sequestration via oranges can be attributed to PLC in the LCA. Coupling this effect with the fact that PLC production utilises CO_2_ as a direct feedstock to the process, PLC acts as a carbon sink effectively sequestering over 9 kg of CO_2_ per 1 kg of PLC produced. The AD/CHP plant is considered as “carbon neutral“ since the CO_2_ released during complete combustion is in balance with the CO_2_ initially captured via photosynthesis.

*Environmental hotspot analysis*. Figure 15 shows the relative contributions of the constituent parts of the PLC process to the midpoint analysis categories. The significant saving effects of CCU is sufficient to offset the environmental burdens incurred by H_2_O_2_ on global warming, leading to PLC with environmental benefits (demonstrated by the violet bar below the x‐axis). Similarly, the relative environmental benefits of implementing resource recovery on climate change impact category are shown by the red bars below the x‐axis.

Two dominating contributors to the environmental impacts throughout the cradle‐to‐gate PLC life cycle were the H_2_O_2_ required, and limonene production process. Despite H_2_O_2_ being highlighted as a potential environmentally beneficial substitute to the previously used TBHP,^10^ this study highlighted the environmental benefits of further research on other sustainable alternatives for oxidising agents or technology development for fossil‐independent H_2_O_2_ production. The H_2_O_2_ process considered in this study relies heavily on energy production from global oil and gas reserves, and the environmental burdens associated with these fossil resources propagate along the supply chain.

To assess the underlying processes or resources causing environmental burdens of limonene production, a hotspot analysis was implemented to the limonene co‐production from the orange juice production process. As presented in Figure 16, WRRS contributed to the environmental benefits, shown as negative values below the x‐axis. The process reliance on electricity provided by the grid and heat from natural gas are demonstrated as one of the contributors sharing 10–20 % of the environmental burdens on climate change, ozone depletion and fossil depletion. However, driven by the agro‐chemical inputs and field operations, orange cultivation was shown as the dominating factor, accounting for 60–90 % of positive scores across all impact categories. At cradle‐to‐farm‐gate stage, the life cycle impacts of producing the processing grade oranges were driven by the nitrogen fertiliser applications and diesel combustion for operating agricultural machinery. Such environmental burdens were attributable to the fossil resource extraction (e. g. natural gas and heavy fuel oil) and atmospheric emissions released (e. g. PAH, NH_3_, N_2_O, CBrClF_3_) from N fertiliser or diesel manufacturing and their supply chains (e. g. NH_3_ production, infrastructure inputs, crude oil production, natural gas transport). This coupled with the large amount of orange feedstock (336 kg) required for limonene processing, results in orange cultivation being the dominating environmental contributor to the PLC manufacturing supply chain. These LCA outcomes highlight sustainable farming strategies as a research frontier for PLC and related technology implementation; notably, precision farming with low energy and agro‐chemical inputs represents a potential solution.

### 4.3 Allocation Method Sensitivity Analysis

**Figure 17:**
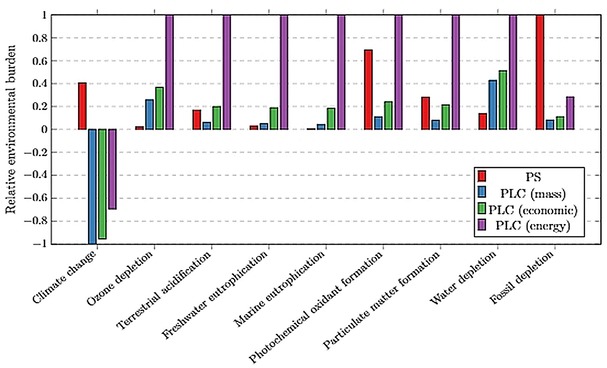
Sensitivity of the ReCiPe Midpoint analysis of the designed PLC production process relative to the allocation method used, per kg polymer produced. PS production is shown for comparison and the PLC process shown is including the WRRS. Scores 0 to 1 represent environmental burdens whilst bars below 0 represent environmental saving effects.

As shown in Table 3, the mass allocation method allocates the lowest fraction of environmental burdens to the limonene (0.3 %), with economic allocation increasing this allocation 10‐fold, and energy allocation increasing even further (to 7.8 %). These allocation procedures allocate a proportion of the environmental burdens of the orange juice production process to limonene. Figure 17 compares the sensitivity of the PLC midpoint analysis with respect to these allocation methods, with PS production shown for reference. Energy allocation changes the environmental analysis outcomes significantly across all impact categories except climate change and fossil depletion. Economic allocation increases PLC environmental burdens across all impact categories, compared to mass allocation, with particularly large increases in acidification and eutrophication categories (3.2‐ to 4.2‐fold increases). These impact categories were highlighted in Figure 16 as being primarily attributed to orange cultivation which is the main contributor to the orange juice production environmental burdens being allocated.

Other impact categories are less sensitive to the allocation method with the PS/PLC comparison being unaffected in most cases. However, PLC exhibits greater environmental burdens in the terrestrial acidification and photochemical oxidation categories when the allocation method shifts from mass to economics, highlighting the importance of underlying LCA methods in informing decision‐making.

### 4.4 Planetary Boundary Considerations

The planetary boundary (PB) framework was introduced to consider the criticality of different planetary systems and define safe human operating boundaries to maintain a stable planet environment.^24^, ^25^ The planetary boundary for atmospheric CO_2_ concentrations is 350 ppmv but we have already transgressed this with a current concentration of greater than 400 ppmv.^24^, ^25^ The organic carbon utilisation coupled with the use of CO_2_ as feedstock in the proposed PLC production process, and similar biopolymer from waste pathways, can help slow the acceleration of atmospheric CO_2_ concentration towards this PB. However, the PB for nitrogen and phosphorus systems should also be mentioned, since orange cultivation was highlighted as a major contributor to the environmental impacts discussed. The proposed biopolymer pathway is certainly a desirable pathway for societal development, especially with regards to climate change mitigation, but upstream industries require environmental optimisation before such a process is implemented or intensified.

The CCU potential of the PLC process (accounting for organic carbon utilisation and CO_2_ immobilised as direct feedstock) is about 9 kg CO_2_ equivalent sequestered per kg of PLC produced. If similar biopolymer processes substitute all standard plastic (Polyolefins, PVC, PS, EPS & PET) production processes (about 285 Mtonne/yr production),^1^ then this CCU potential increases to about 2.6 Gtonne/yr CO_2_ equivalent or a reduction in atmospheric carbon concentration of about 0.33 ppm/yr. The 2016–2017 growth in atmospheric CO_2_ concentration was 2.14 ppm/yr,^36^ so in this scenario, these biopolymer pathways could reduce this growth rate by about 15 % ‐ a conservative estimate not accounting for the PS CO_2_ emissions that would be displaced.

## 5 Discussion and Conclusion

This study bridges the emerging research gaps on techno‐economic and environmental viability of a closed‐loop manufacturing system to co‐produce a biopolymer and energy, where a novel synthetic pathway for agricultural waste‐derived PLC and CCU technologies have been modelled. This research highlights the techno‐environmental potential of PLC production systems to mitigate climate change by fixing the biogenic carbon through photosynthesis in biomass‐derived PLC and capturing carbon through copolymerisation. Specifically, this study adopts a holistic approach to the process design, considering technical, economic and environmental perspectives. Our modelling results suggest that a process to produce 21 ktonne/yr of PLC is technically feasible with an economic potential of about 21.4 M USD/yr. From an environmental sustainability perspective, the PLC system offers superior environmental profiles to PS production in the climate change and fossil depletion impact categories. However, it does not compare well with conventional PS counterparts with regards to eutrophication, water consumption and ozone depletion.

Process analysis determined that the annualised expenditure on such a PLC process would be 20.6 M USD/yr, with an associated revenue of 32.7 M USD/yr (excluding any utility sales). It was found that the largest two contributors to the overall costs were the separations and WRRS. The high capital costs of WRRS, that account for 53 % of the total costs, are due to the large mass flows of wet citrus waste remaining after the extraction of limonene.

Future research should address the limitations in the process design section of this study to ensure that the assumptions made do not compromise the economic or environmental analysis: first, side reactions in the epoxidation reactor should be considered, necessitating the incorporation of additional chemicals such as sodium sulphate to prevent hydrolysis of the epoxide;^10^ second, catalyst separation and recycling costs should be considered for more complete flowsheet design.

The PLC production system designed in this study is resilient to the operational risks caused by potential market fluctuations (e. g. PLC price, supply‐demand balances, seasonal supply fluctuations) and risks in orange supply caused by extreme events. The techno‐economic analyses demonstrate that 35 % of the annual operating capacity is the threshold for the PLC biorefinery to be profitable. This increases the confidence in design robustness considering large feedstock demands by the biorefinery, which is equivalent to 40 % of São Paulo's annual supply.

This study also demonstrates that the economic feasibility of PLC is sensitive to plant location. Similarly, the LCA results also depended on location due to the difference in energy mix of different countries’ grids. Since large quantities of citrus waste were required to produce the desired amount of PLC, it was important to establish the refinery in a region with a concentrated citrus industry to avoid dependence on feedstock imports, which may have several consequences: increasing the logistics complexity; causing environmental burdens due to freight transport emissions; and increasing economic risk due to feedstock decomposition and long‐distance transportation.

The energy consumption of the process was optimised by minimising the separation costs, which account for 26 % of the total annualised costs for the process. This is mostly due to the two distillation columns that are required to separate limonene and limonene oxide (C3 and C4). These two columns require high reflux ratios and many stages to achieve the desired degree of separation for two reasons: first, the two components have very similar boiling points; second, it is important that the limonene oxide product stream from the epoxidation reactor loop achieves a high purity. Since limonene is inert in the polymerisation reaction loop, any impurities would build up, increasing its concentration in the waste streams. Therefore, two large distillation columns were assumed, to ensure purity and recovery of limonene oxide in the bottom product of the separation sequence.

The energy source has a significant contribution to the environmental profiles of PLC, where the beneficial effects associated with substituting fossil dependent energy production with energy from biowaste are highlighted. In this study, a WRRS scenario was modelled with the generation of 100 MW of excess energy from the citrus waste. This reduced environmental burden scores across all impact categories, by up to 23 %, sensitive to allocation method used. When analysing the optimum operating conversion for the process, it was observed that the refinery shifted the dominant product from PLC to energy generation.

H_2_O_2_ is highlighted in our analyses as one of the major contributors to the environmental impact of the PLC production lifecycle, notably contributing 50–85 % of the environmental burdens in ozone depletion, photochemical oxidation and PM formation. Furthermore, H_2_O_2_ raises safety concerns due to its rapid decomposition into oxygen and water at high temperatures. To carry out separation at lower temperatures, vacuum distillation columns were implemented which further added to the costs of separation. Additionally, due to handling considerations, H_2_O_2_ comes as a 50 wt % solution with water, which greatly increases the mass flows that are required to go through the water separation column and adds to the negative impact of the process in terms of water depletion. Our results highlight alternative sustainable oxidising agents as useful research directions in terpene oxidation.

In addition, farming practices are suggested as another factor hindering the sustainable production of PLC; orange cultivation represented the second major contributor to the environmental impacts throughout the PLC cradle‐to‐gate life cycle due to the fertiliser inputs and diesel‐powered agricultural machinery. The environmental burdens associated with extracting and processing these fossil reserves, coupled with the large amount of mass requiring processing upstream (due to the low mass concentration of limonene within oranges), results in significant environmental burdens propagating along the PLC production chain. The hotspot analysis highlighted that there is a clear need for cross sector collaboration to shift towards a low fossil‐input biopolymer refinery system which has the potential to impact our sustainable future.

To improve the economic and environmental performance of the process, our results suggest to consider alternative waste resource recovery pathways. In our study we exhibited the environmental benefits of implementing an AD treatment to cogenerate energy but this comes with a high capital cost, accounting for 53 % of total costs. Future research into alternative and integrated resource recovery technologies has the potential to realise similar (or greater) environmental benefits at a potentially reduced total cost.

The contribution of the proposed PLC process, and similar biopolymer pathways, to achieving sustainable practices in reference to planetary boundaries was explored. Such carbon‐sequestering bioplastics could slow the escape of atmospheric CO_2_ concentrations from the planetary boundary by 15 % if all standard petroleum‐based plastic production processes were substituted. This highlights the importance of biopolymer research and integration into future sustainable bioeconomy visions.

## Conflict of interest

The authors declare no conflict of interest.

## Supporting information

As a service to our authors and readers, this journal provides supporting information supplied by the authors. Such materials are peer reviewed and may be re‐organized for online delivery, but are not copy‐edited or typeset. Technical support issues arising from supporting information (other than missing files) should be addressed to the authors.

Supplementary
