# Supplementary material for: Scale‐up and Sustainability Evaluation of Biopolymer Production from Citrus Waste Offering Carbon Capture and Utilisation Pathway
Source: ChemistryOpen. 2019 Mar 7;8(6):668–88. doi: 10.1002/open.201900015 (PMC6547945; doi:10.1002/open.201900015)
Supplement: Supplementary file 1 — Supplementary [file OPEN-8-668-s001.pdf]

Open Access
ChemistryOPEN

Supporting Information

© Copyright Wiley-VCH Verlag GmbH & Co. KGaA, 69451 Weinheim, 2019

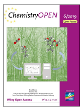

Scale-up and Sustainability Evaluation of Biopolymer Production from Citrus Waste Offering Carbon Capture and Utilisation Pathway

Alex Durkin, Ivan Taptygin, Qingyuan Kong, Mohamad F. M. Gunam Resul, Abdul Rehman, Ana M. L. Fernández, Adam P. Harvey, Nilay Shah,* and Miao Guo*© 2019 The Authors. Published by Wiley-VCH Verlag GmbH & Co. KGaA.
This is an open access article under the terms of the Creative Commons Attribution License, which permits use, distribution and reproduction in any medium, provided the original work is properly cited.

Supplementary Information
SI-1 Level 1: Mode of operation

To decide on the mode of operation the following factors were considered:

Production rate.
The desired production rate of PLC is 4.76x10^7 lb/yr which is larger than the recommended upper limit of 1x10^7 lb/yr for a batch process suggested by Douglas. This suggests that continuous mode of operation is preferable to manage the large throughput of mass in the system. Furthermore, the higher costs of continuous process equipment are justified by the higher revenues gained from the large production rate. However, polymers are usually produced using batch reactors. In this study, the process was modelled as continuous but it is worth noting that for a more detailed process design, batch reactors should be considered.

Market forces.
PLC is comparable to PS in its physical properties and applications, hence it has a very wide range of possible sources of demand, ranging from producing plastic cutlery to CD cases. The global demand for PS is non-seasonal and is increasing each year. Therefore, a continuous process would suit this market better since it will be able to better accommodate and adjust for the continuous demand for PLC whilst also making it easier to expand to meet increasing demand. Furthermore, the citrus waste required for limonene production can be obtained from processing grade oranges, that have very little seasonal fluctuation in supply. This is because the plant is designed to be in a location, close to large orange juice production facilities, and can use the waste and inedible oranges as feedstock. This further supports the choice of a continuous process.

Practicality.
The PLC production rate that is considered is about 2.7 tonnes/hr, which is very large. This suggests that, assuming transport processes are designed sufficiently to handle solids, there should not be any problems with plugging of the equipment. Furthermore, PS plants with comparable production rates use continuous production processes, suggesting that it is practically feasible to use a continuous process to produce PLC with very few potential problems.

SI-2 Level 2: input-output structure

Feed stream purification.
H_2O_2 is available at 50 wt% whilst all other reactants are assumed to be pure. According to Douglas heuristics, it is better to purify a liquid stream containing high amounts of impurities (which is also a by-product of the reaction) upstream of the reactor. Therefore, vacuum distillation is required due to safety hazards associated with H_2O_2.

Recover or recycle reversible by-products.
In this process, the only by-product is water, which is produced in Reaction 1. The reaction is not reversible so there is no need to recycle the water that is produced. It should instead be separated from the product stream.

Gas recycle and purge.
Since the only gas stream in the process is the CO_2 feed to the second reactor, any unreacted CO_2 should be recycled back to the feed. There are no gaseous by-products that will build up in the gas recycle loop and accumulation of CO_2 in the system can be prevented by implementing feed controllers.

Recover and recycle.
In this process, the recovery of the valuable materials including limonene, H_2O_2, limonene oxide, CO_2 and PLC needs to be considered. Furthermore, it is important that limonene oxide and PLC are recovered in pure

forms. It is assumed that PLC is sold to market at >99% purity levels, same as PS. Limonene oxide should be purified to ensure that components from Reaction 1 do not contaminate Reaction 2, since they would be inert in the Reaction 2 system and therefore likely to build up. Water is much less valuable; however, it could be used in other parts of the plant for heat transfer or as a safety control for H_2O_2 concentrations in the process. The water is therefore separated out of the system, but little effort is put into purifying it. Waste treatment is considered in Level 4 of the Douglas design framework.

SI-3 Aspen flowsheet

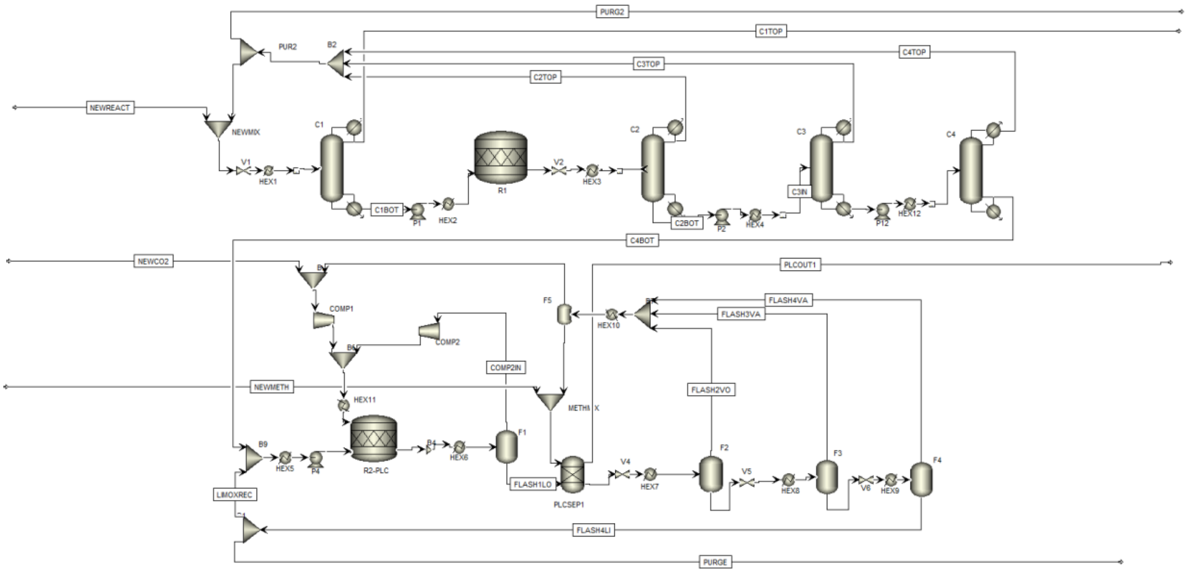

Figure S1: Aspen flowsheet for PLC production process.

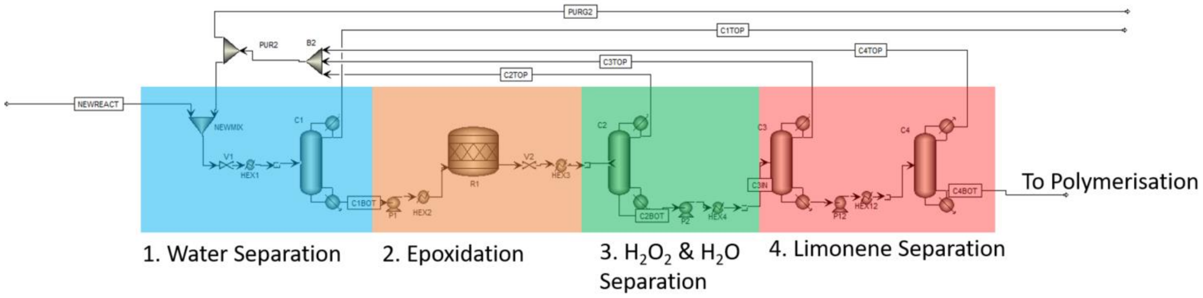

Figure S2: Labelled Aspen flowsheet for limonene oxide synthesis.

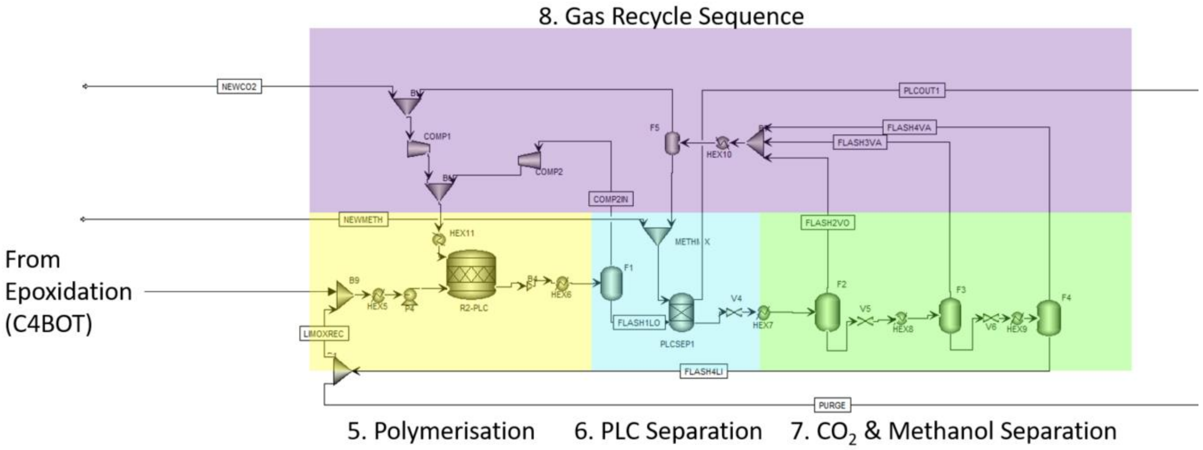

Figure S3: Labelled Aspen flowsheet for polymerisation pathway.

SI-4 Summary of Douglas Design Levels

Table S1: Description of Douglas design levels.

Douglas Level	Key Decisions	Key Considerations	Assumptions	Economic Potential Equation
1	Mode of Operation	Production Rate<br>Market Forces<br>Operational Problems	100% Conversion<br>Perfect Separation<br>Pure Feeds<br>No Side Reactions<br>Stoichiometric Reactant Feed<br>Non-Degradable Catalyst	$EP_1 = Revenue_{PLC} - Cost_{reactants}$
2	Input-Output Structure	Feed Stream Purification<br>Recycle of By-Products<br>Gas Recycle & Purge<br>Component Destinations	Perfect Separation<br>Stoichiometric Reactant Feed<br>Non-Degradable Catalysts	$EP_2 = EP_1 + Revenue_{side\ products} - Cost_{waste\ processing}$
3	Recycle Structure	Reactor System Structure<br>Detailed Recycle Structure<br>Reactor Heat Effects & Costs<br>Compressor Cost	Perfect Separation<br>Non-Degradable Catalysts	$EP_3 = EP_2 - (Annualised\ Cost\ of\ Reactors + Annualised\ Cost\ of\ Compressor)$
4	Separation System	Jacksland et al. Analysis<br>Douglas Separation Heuristics<br>Thermodynamic Method<br>Waste Disposal Method<br>Feasible Design Alternatives	Non-Degradable Catalysts	$EP_4 = EP_3 - Separation\ Cost - Heat\ Exchanger\ Cost$
5	Heat Integration & Additional Considerations	Composite Curve Analysis<br>12 Principles of Green Engineering<br>Process Safety<br>Process Control	Minimal driving force for heat exchangers = 10K<br>All heat exchanger systems are adiabatic<br>Non-Degradable Catalysts	$EP_5 = EP_4 + Heat\ Integration\ Savings$

SI-5 Data for epoxidation reaction and kinetics calculation

The reaction kinetics for the epoxidation reaction were assumed to be first order w.r.t. limonene and hydrogen peroxide. This means that the following rate equation holds true:

$$H_2O_2 = A;\ Limonene = B$$
$$r_A = k[A][B]$$
$$\frac{1}{V}\frac{dN_A}{dt} = k[A][B]$$
$$[A][B]V = \frac{N_A N_B}{V}$$
$$\frac{dN_A}{dt} = k\frac{N_A N_B}{V}$$

This can be approximated by the following equation:

$$\frac{\Delta N_A}{\Delta t} = k\ \frac{N_{A,Average}N_{B,Average}}{V}$$

Where the average N_A and N_B are calculated to match with the rate of reaction at a specific time. Therefore, plotting the approximated rate against the $\frac{N_{A,Average}N_{B,Average}}{V}$ term, gives the approximated k value as the gradient. The tables and graphs below show calculations using empirical data from Resul et al.:

Table 2: Epoxidation kinetics calculations.

MR = 1							
Time / min	H2O2 conversion / %	N (H2O2) / mol	N (lim) / mol	dN (H2O2) / mol	dN/dt / mol/s	N(H2O2)*N(lim)/V / mol^2/m^3	Average to match rate
0	0.00	0.12	0.12	-	-	2.98E+03	-
5	53.15	0.06	0.18	0.06	0.000216	2.05E+03	2512
10	71.22	0.04	0.16	0.02	7.35E-05	1.10E+03	1576
15	85.53	0.02	0.14	0.02	5.82E-05	4.93E+02	798
30	85.90	0.02	0.14	0.00	5.09E-07	4.79E+02	486
MR = 2							
Time / min	H2O2 conversion / %	N (H2O2) / mol	N (lim) / mol	dN (H2O2) / mol	dN/dt / mol/s	N(H2O2)*N(lim)/V / mol^2/m^3	Average to match rate
0	0.00	0.12	0.24	-	-	5953.60	-
5	65.89	0.04	0.16	8.04E-02	2.68E-04	1361.94	3658
10	86.21	0.02	0.14	2.48E-02	8.26E-05	467.13	915
15	95.10	0.01	0.13	1.08E-02	3.62E-05	152.99	310
30	97.17	0.00	0.13	2.53E-03	2.81E-06	86.58	120

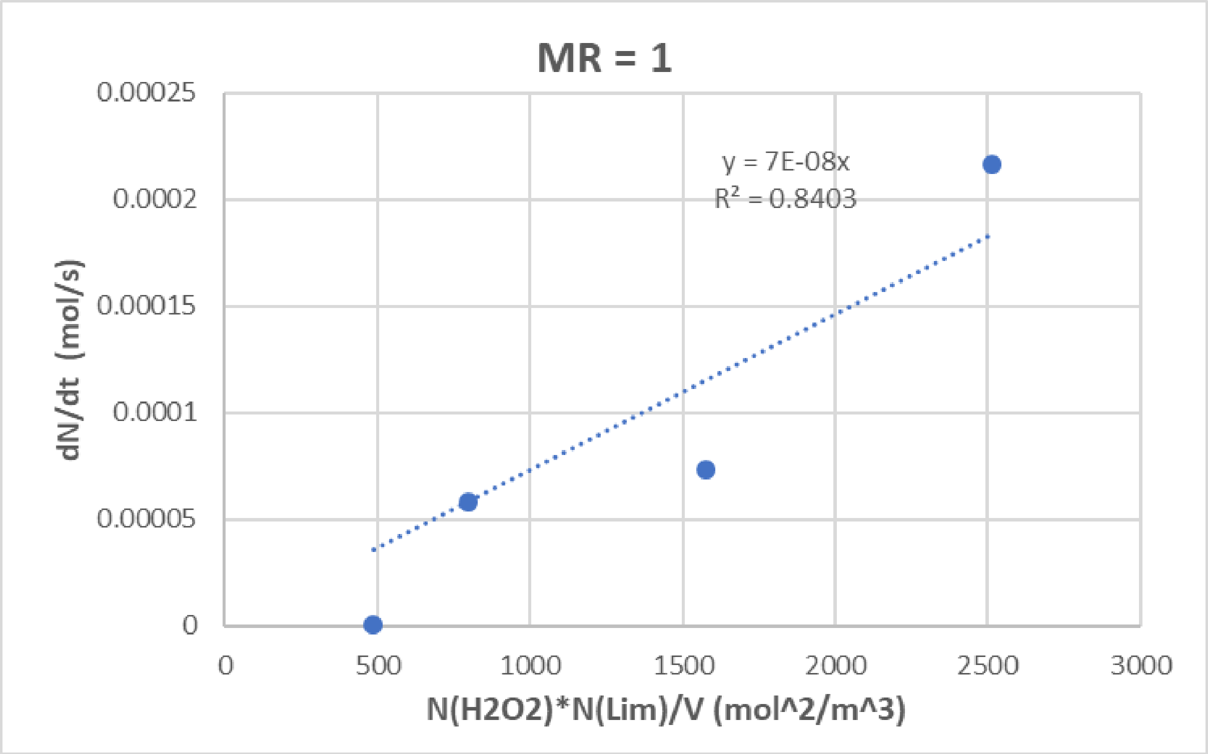

Figure S4: Linearisation of epoxidation kinetics at molar ratio limonene:hydrogen peroxide equal to 1.

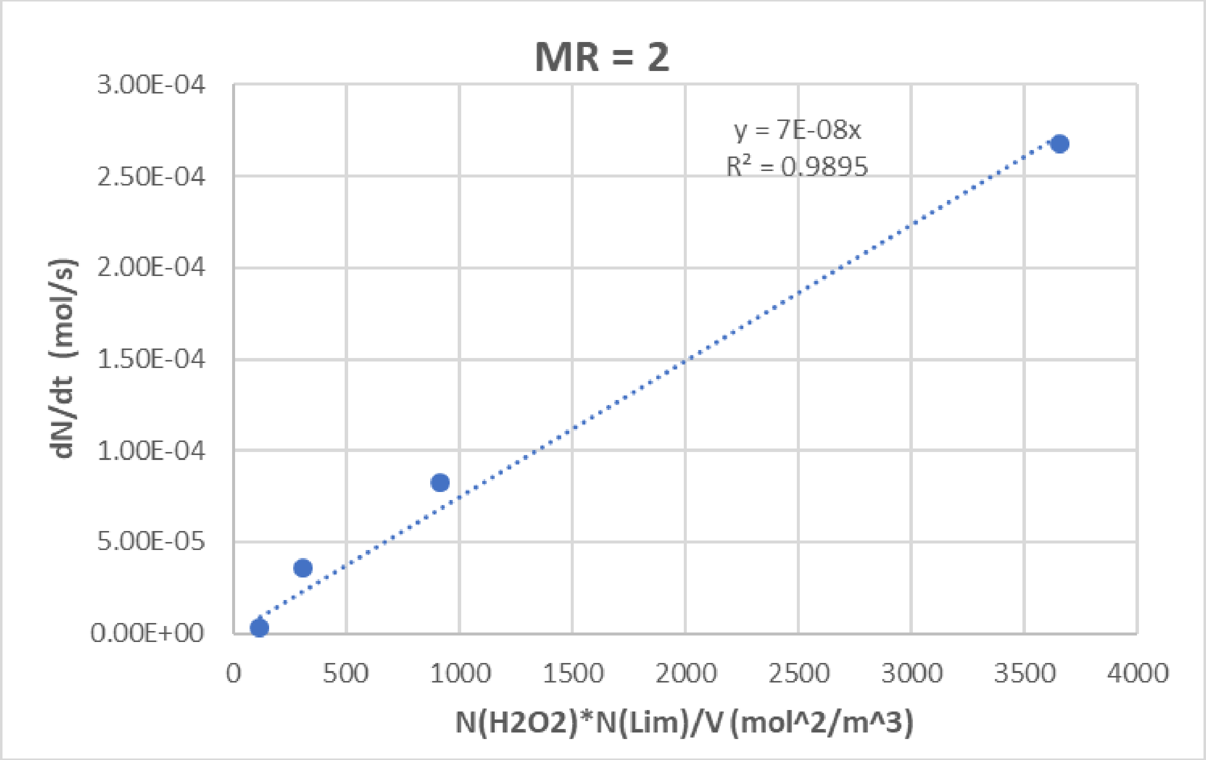

Figure S5: Linearisation of epoxidation kinetics at molar ratio limonene:hydrogen peroxide equal to 2.

The volume of the laboratory reactor was given as 5 x 10^{-6} m^3. Due to the uncertainty of the results for MR = 1, the k value was taken to 1 significant figure as 7 x 10^{-8} m^3 mol^{-1} s^{-1}.

SI-6 Limonene Extraction Costs Estimation

It was assumed that the main costs for limonene extraction are the variable costs involved in the hydrolysis process. It was found that the reaction conditions of the hydrolysis reactor are 150°C and the reactor has a residence time of 6 minutes. Assuming that the main cost of the process arises from using steam to heat up the citrus waste mass to 150°C. The average heat capacity of citrus waste was assumed to be similar to other biomass heat capacity. To get an accurate estimate for the heat capacity, values for other biomass types were averaged:

Table S3: Biomass heat capacities to calculate average for limonene.

Biomass	Heat capacity / J/kg.K
Wheat	1336
Rice	1377
Olive	1345
Corn	1395
Average	1363

It was determined that for every 2.26kg of limonene produced in the hydrolysis reactor, 168kg of citrus waste is processed. Therefore, 74.3 kg of citrus waste per kg of limonene has to be heated up to 150°C (302°F). Assuming that waste enters at 25°C:

$$Q = C_{p,CW}\Delta T$$

$$Q = 1363 \times 125 = 170.3\frac{kJ}{kg(CW)}$$

$$= 170.3\frac{kJ}{kg(CW)} \times 74.3\frac{kg(CW)}{kg(limonene)} = 12659\frac{kJ}{kg(limonene)}$$

To price the extraction process, saturated steam at 487°F and 600psig (priced at 0.00452 $/lb) was used. The latent heat for this steam is 713.4 BTU/lb.

$$12659\frac{kJ}{kg(limonene)} \times \frac{1\ BTU}{1.055\ kJ} = 12000\frac{BTU}{kg(limonene)}$$

$$\therefore 12000\frac{BTU}{kg(limonene)} \times \frac{1\ lb}{713.4\ BTU} \times 0.00452\frac{\$}{lb} = 0.076\frac{\$}{kg(limonene)} = \mathbf{76}\frac{\$}{\mathbf{metric\ tonne}}$$

SI-7 Epoxidation Reactor Simulation – MATLAB Code

To determine reactor heat effects, size and costs, a model was compiled in Matlab. The code used is below:

```
function  [A] = LOreactor(it)

% initialise
A = zeros(it,2);
V = zeros(it);
xvec = zeros(it);
i = 0;

stepsize = 1/(it-1);

for x = 0:stepsize:1

    i = i + 1;
    xvec(i) = x;

    % molecular mass
    MW_H2O2 =   34*10^-3;        % molecular weight H2O2, kg/mol
    MW_H2O  =   18*10^-3;        % molecular weight H2O, kg/mol
    MW_Lim  =   136.24*10^-3;    % molecular weight limonene, kg/mol
    MW_LO   =   152.24*10^-3;    % molecular weight limonene oxide, kg/mol

    % densities ( assume constants )
    rho_H2O2    =   1404;        % hydrogen peroxide density, kg/m3
    rho_H2O     =   983.2;       % water density, kg/m3
    rho_Lim     =   820;         % limonene density, kg/m3
    rho_LO      =   820;         % limonene oxide density, kg/m3

    % inlet stream compositions
    MR      =   2;               % molar ratio of Limonen:H2O2
    w_H2O2  =   0.6;             % mass fraction of H2O2 in H2O2 / water feed
    x_H2O2  =   (w_H2O2 / MW_H2O2) / ((w_H2O2 / MW_H2O2 ) + ((1 - w_H2O2) /
MW_H2O) );
                                 % conversion of mass fraction to mole fraction

    % inlet flows
    nH2O2_i =   3.83 / x;        % mol/s
    nLim_i  =   nH2O2_i*MR;      % mol/s
    nLO_i   =   0;               % mol/s
    nH2O_i  =   nH2O2_i * ( 1 - x_H2O2 ) / x_H2O2;  % mol/s

    % outlet flows
    nLim    =   nLim_i - nH2O2_i*x;      % unreacted limonene, mol/s
    nH2O2   =   nH2O2_i - nH2O2_i*x;     % unreacted hydrogen peroxide, mol/s
    nLO     =   nLO_i + nH2O2_i*x;       % limonene oxide produced in reaction 1,
mol/s
    nH2O    =   nH2O_i + nH2O2_i*x;      % water inert in system, mol/s
    n       =   [ nLim nH2O2 nLO nH2O ];

    % volumetric flowrate
    vt   =   nLim*MW_Lim/rho_Lim + nH2O2*MW_H2O2/rho_H2O2 + nLO*MW_LO/rho_LO +
nH2O*MW_H2O/rho_H2O;
                                 % total vol flowrate at outlet conditions

    % vector of concentration
    c    = n./vt;                % vector of concentrations [ cLim cH2O2 cLO cH2O
], mol/m3

    % reaction kinetics
```

```
    k    = 7*10^-8;                 % k value, m3/mol.s

    % reaction equation
    dVdx    =    nH2O2_i/(k*c(1)*c(2));    % gradient of V against x

    % generate plotting vectors
    if i == 1
        v = 0;
    else
        v = V(i-1) + (xvec(i)-xvec(i-1)) * dVdx;
    end

    V(i) = v;

    A(i,:) = [ x,V(i) ];

end

% make plots
plot(A(:,1),A(:,2))
axis([0.2 0.9 0 100])

xlabel('Conversion')
ylabel('R1 volume (m^3)')
title('Volume against conversion in R1')
```

This code produced a relationship between the conversion of epoxidation reaction and its residence time, as seen below:

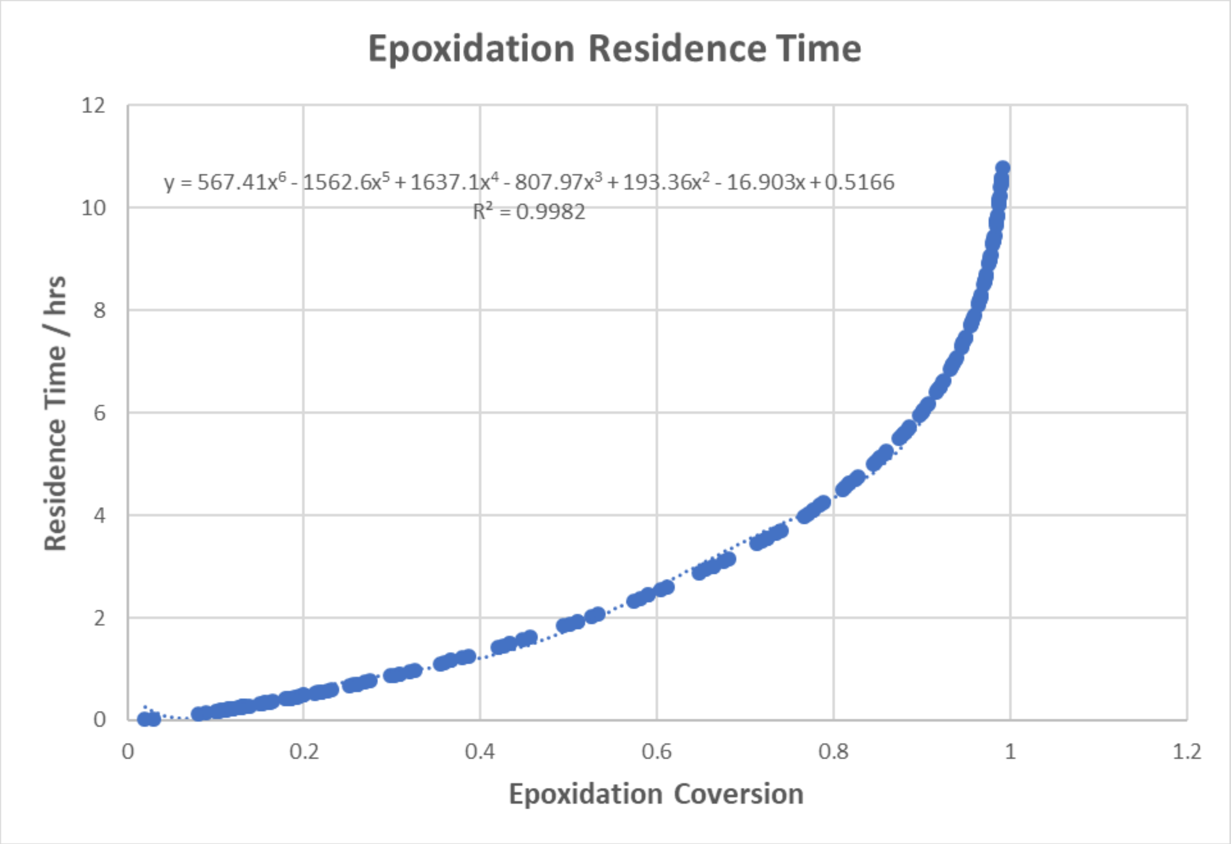

Figure S6: Residence time vs conversion for epoxidation reactor modelled in MATLAB.

This therefore allowed the calculation of reactor costs at different conversions.

SI-8 Process Units Costs Calculations

Installed Costs

Table S4: Correlations for installation costs for process units.

Process Unit	Gunthrie's Correlation (Note: It was assumed that prices of flash vessels were negligible)	Supplementary Equations
Reactor	$Annualised\ Installation\ Cost\ (\$) = \frac{1}{3} \times \frac{M\&S}{280} \times 101.9 D^{1.066} L^{0.802} (F_{c,reactor} + 2.18)$	$D_R = \left(\frac{4V_R}{\pi R}\right)^{\frac{1}{3}}$ $L_R = (D_R R) = R\left(\frac{4V_R}{\pi R}\right)^{\frac{1}{3}} = R^{\frac{2}{3}}\left(\frac{4V_R}{\pi}\right)^{\frac{1}{3}}$ $V_R = \frac{v_t}{\tau}$ R, Aspect Ratio of Reactor = 6
Compressor	$Annualised\ Installation\ Cost\ (\$) = \frac{1}{3} \times \frac{M\&S}{280} \times 517.5(bhp)^{0.802} (F_{c,compressor} + 2.11)$	$bhp = \frac{1}{0.9}\left(\frac{3.03 \times 10^{-5}}{\gamma}\right) P_{in} Q_{in} \left[\left(\frac{P_{out}}{P_{in}}\right)^{\gamma} - 1\right]$
Heat Exchanger	$Annualised\ Installed\ Cost\ (\$) = \frac{1}{3}\left(\frac{M\&S}{280}\right) 101.3 A^{0.65} (2.29 + F_{HEX})$	$A_{HE}[ft^2] = \left\| \frac{Q_{HD}\left[\frac{Btu}{hr}\right]}{\lambda \left[\frac{Btu}{hr.ft^2.F}\right] \times \Delta T_{lm}[F]} \right\|$ $\Delta T_{lm}(F) = \frac{(T_{1,in} - T_{2,in}) - (T_{1,out} - T_{2,out})}{\ln\left(\frac{T_{1,in} - T_{2,in}}{T_{1,out} - T_{2,out}}\right)}$
Distillation Column	$Annualised\ Installed\ Cost_{Column\ shell}(\$) = \left(\frac{1}{3}\right)\left(\frac{M\&S}{280}\right) 101.9 D_c^{1.066} H_{shell}^{0.802} (2.18 + F_{c,S})$ $Annualised\ Installed\ Cost_{Column\ Trays}(\$) = \left(\frac{1}{3}\right)\left(\frac{M\&S}{280}\right) 4.7 D_c^{1.55} H_{Stack} F_{c,T}$ Reboiler and condenser priced same as other heat exchangers	$A_C[ft^2] = 2.1 \times 10^{-4} V_{max}[mol\ hr^{-1}] \left(\frac{M_G[lb/mol]}{\rho_m[mol/ft^3]}\right)^{\frac{1}{2}}$ $D_c[ft] = \sqrt{\frac{4A_T[ft^2]}{\pi}}$ $H_{stack}[ft] = 2ft \times No.of\ Stages$ $H_{Shell}[ft] = H_{Stack}[ft] + 15ft$
Vacuum Pump	The pumping costs depend on the volumetric flowrate of gas (S $\left[\frac{m^3}{h}\right]$) through them such that:	

	When S > 5000$\frac{m^3}{h}$, Blower, 1-Stage Liquid Seal is used, hence:<br>$Installed\ Cost\ of\ Vacuum\ Pump(\$) = \ 8.0882\left[\frac{\$.hr}{m^3}\right] \times S\ \left[\frac{m^3}{hr}\right]\ + 19137[\$]$<br>When S < 5000$\frac{m^3}{h}$, Blower, 1-Stage Dry Seal is used, hence:<br>$Installed\ Cost\ of\ Vacuum\ Pump\ (\$) = \ 19.615\left[\frac{\$.hr}{m^3}\right] \times \ S\left[\frac{m^3}{hr}\right] + 17576[\$]$<br>The annualized costs are calculated as follows:<br>$Annualised\ Installed\ Cost\ of\ Vacuum\ Pump(\$) = \frac{1}{3} \times Installed\ Cost\ of\ Vacuum\ Pump\ [\$]$	

The $F_{c,i}$ factors depend on the type of material that is used for the process equipment, as well as the operating pressure of the equipment:

$$F_{c,i} = F_{m,i}F_{p,i} = 3.67\ \times 1.05 = 3.85$$

Solid SS 316 was selected as the material of choice, due to the presence of H_2O_2. H_2O_2 is an excellent oxidising agent, meaning that it is likely to increase the rate of corrosion in the equipment. To minimise this, stainless steel was selected as the material. The polymerisation reactor has an operating pressure of 7 bar, which is just over 100 psig, hence 1.05 was chosen as F_p.

The material factors are calculated differently for heat exchangers:

$$F_{HEX} = \left(F_{d,HEX} + F_{p,HEX}\right)F_{m,HEX}$$

Furthermore, depending on the layout and the type of heat exchanger, different overall heat transfer coefficients need to be used:

Table S5: Process unit heat transfer coefficients.

Heat Exchanger Type	Overall Heat Transfer Coefficient Expression
Condenser	$\lambda_{CD} = 100\ \frac{Btu}{hr.ft^2.F}$
Reboiler	$\lambda_{RB}\ \Delta T_{lm} = \ 11{,}250\frac{Btu}{hr.ft^2}$
Other HEXs	$\lambda_{HEX} = 50\left[\frac{Btu}{hr\ ft^2\ F}\right]$

Table S6: Gutherie material and pressure factors for pressure vessels: MPF =Fm Fp

Shell Material	Clad, Fm	Solid, Fm					
Carbon Steel (CS)	1.00	1.00					
Stainless 316 (SS)	2.25	3.67					
Monel (Ni:Cr/2:1 alloy)	3.89	6.34					
Titanium	4.23	7.89					
Vessel Pressure (psig)							
Up to	50	100	200	300	400	500	900	1000
Fp	1.00	1.05	1.15	1.20	1.35	1.45	2.30	2.50

Table S7: Correction factors for process units.

Correction factor	Fm	Fd	Fp
Condenser	3.75 (SS)	0.85 (U-tube)	0
Reboiler	3.75 (SS)	1.35 (Kettle)	0
Other HEX	3.75 (SS)	0.85 (U-tube)	0

Lang Factor

To estimate the other costs that are associated with the equipment, the Lang Factor approach is used. It is possible to calculate the Lang Factor, using approximated fractions of costs associated with installing the equipment (e.g. scaffolding costs, contractor costs, land costs, etc.). Since it is difficult to get hold of accurate accounting data for process plants, a value of 4 was taken (Wolf, 2013). Whilst literature suggested a value of 3.63, it was decided to round this value up to 4, since the proposed process is novel and hence is likely to experience unforeseen costs.

Example Calculations

Since a distillation column is able to cover most of the equipment costs (shell cost, tray cost, reboiler, condenser and a vacuum pump), an example calculation for one is presented below. This is done for a conversion of 0.8 on C1. Since the process was extensively modelled in Aspen, it was possible to gather most of the required information from the simulation outputs.

Shell and Tray Costs

$$\frac{M_G}{\rho_m} = 0.0059; V_{max} = 10500\frac{mol}{hr}; No.\,of\ Stages = 10$$

$$A_C = 2.1 \times 10^{-4} \times 10500 \times (0.0059)^{\frac{1}{2}} = 1.69 ft^2$$

$$\therefore D_c = \sqrt{\frac{4 \times 1.69}{\pi}} = 1.47\ ft$$

$$H_{stack} = 2ft \times 10 = 20\ ft$$

$$\therefore H_{Shell} = 20 + 15 = 35ft$$

$$Annualised\ Installed\ Cost_{Shell} = \left(\frac{1}{3}\right)\left(\frac{1445}{280}\right)101.9 \times 1.47^{1.066} \times 35^{0.802}(2.18 + 3.85)$$

$$= \$\ 27500/yr$$

$$Annualised\ Installed\ Cost_{Trays} = \left(\frac{1}{3}\right)\left(\frac{1445}{280}\right)4.7 \times 1.47^{1.55} \times 20 \times 3.85 = \$1130/yr$$

Reboiler Cost

$$Q_{RB} = 1920\frac{MBTU}{hr}; T_{1,in} = T_{1,out} = 199°\text{F}, T_{2,in} = T_{2,out} = 487.4°\text{F}$$

$$\therefore\ \Delta T_1 = \Delta T_2 = 288.4°\text{F};\ \Delta T_{lm} = 288.4°\text{F}$$

$$A_{HE}[ft^2] = \left|\frac{1920 \times 10^3}{11{,}250}\right| = 170.7\ ft^2$$

$$Annualised\ Installed\ Cost\ (\$) = \frac{1}{3}\left(\frac{1445}{280}\right) 101.3 \times 170.7^{0.65}(2.29 + 5.06) = \$36\ 200/yr$$

Vacuum Pump

$$S = 1710 \frac{m^3}{hr}$$

$$Installed\ Cost\ of\ Vacuum\ Pump\ (\$) = 19.615 \times 1710 + 17576 = \$51\ 100/yr$$

Total Column Cost

The total annualised installed cost of the 5 components of C1 is \$186 500/yr. To estimate the annualised cost of all installation costs and maintenance costs, this figure needs to be multiplied by the Lang Factor. Therefore, the overall annualised cost to be taken away from economic potential is \$745,900/yr.

SI-9 Utility Costs

Table S8: Utility costs.

Utility	Price
Saturated steam at 167F	0.0034 USD/lb
Saturated steam at 487F	0.0045 USD/lb
Cooling water at 68F	3.05 USD/1000m3
Electricity	0.0116 USD/kWh

There are three main utility costs associated with the process: cooling, heating and electricity. The costs for this were calculated using the heating duties and other parameters that were taken from Aspen's output. See Table XX for the utilities that were considered.

Heating Utility Costs

The annual cost of the utility was calculated as follows:

$$Annual\ Utility\ Cost\ [\$] = \frac{Q\left[\frac{BTU}{hr}\right]}{\Delta h_{vap,steam}\left[\frac{BTU}{lb}\right]} \times Utility\ Cost\left[\frac{\$}{lb}\right] \times 8000 \frac{operating\ hrs}{year}$$

C1 Reboiler Example Calculation

The heating utility of heat exchangers and reboilers are outputs from Aspen simulation:

$$Q_{C1,RB} = 1922 \frac{MBTU}{hr}; T_{RB} = 199°\text{F}\ \therefore\ steam\ @\ 487°\text{F}\ is\ used$$

$$Annual\ Utility\ Cost\ [\$] = \frac{1922 \times 10^3}{713.4} \times 0.00452 \times 8000 = \$\ 97400/yr$$

Cooling Utility Calculation

The annual cost of the utility was calculated as follows:

$$Annual\ Utility\ Cost\ [\$] = \frac{Q\left[\frac{BTU}{hr}\right]}{\Delta h_{max,water}\left[\frac{BTU}{1000m^3}\right]} \times Utility\ Cost\left[\frac{\$}{1000\ m^3}\right] \times 8000 \frac{hrs}{year}$$

C3 Condenser Example Calculation

$$Q_{C3,CD} = 7610 \frac{MBTU}{hr}; T_{C3,CD} = 273°\text{F}$$

Assuming that cooling water can only be heated up to 122°F (50 C):

$$\Delta h_{max,water} = (122 - 68)[°\text{F}] \times 2.205\left[\frac{BTU}{kg°\text{F}}\right] \times 1000\left[\frac{kg}{m^3}\right] \times 1000\left[\frac{m^3}{1000m^3}\right]$$

$$= 119.0 \times 10^6 \frac{BTU}{1000m^3}$$

$$Annual\ Utility\ Cost\ [\$] = \frac{7610 \times 10^3}{119.0 \times 10^6} \times 3.05 \times 8000 = \$\ 1560/yr$$

Electrical Utility Calculation

The electricity requirement for various pumps was calculated based on the volumetric flowrate through them and the pressure change across them, as follows:

$$Q\ [W] = \Delta P\ [Pa]\ \times\ v_T \left[\frac{m^3}{s}\right]$$

$$Annual\ Utility\ Cost\ [\$] = Q\ [W] \times 3600 \left[\frac{s}{hr}\right] \times 8000 \frac{hrs}{year} \times \frac{1}{3.6\ \times 10^6} \left[\frac{kWhr}{J}\right] \times 0.0116 \left[\frac{\$}{kWhr}\right]$$

C1 Vacuum Pump Example Calculation

$$Q\ = 10^5\ [Pa] \times\ 1710\ \left[\frac{m^3}{hr}\right] \times \frac{1}{3600} \left[\frac{hr}{s}\right] = 47.5\ kW$$

$$Annual\ Utility\ Cost\ [\$] = 47\ 500\ \times\ 3600\ \times\ 8000\ \times\ \frac{1}{3.6\ \times 10^6} \times 0.0116 = \$4410/yr$$

SI-10a Jacksland et al. Analysis

The Jacksland et al. method is outlined below:

1. Collect relevant physical properties for all components in the system
2. Calculate the binary property ratios for all pairs in the system
3. Compare the binary property ratios for each technique k and component i (r_{ik}) with the good (r_{kg}) and feasible ratios (r_{kf}). If r_{ik} is smaller than the feasible ratio, the separation technique can be disregarded.
4. To rank the separation techniques, μ_{ik} is calculated. The higher the μ_{ik} value, the better the separation technique is.

$$\mu_{ik} = \frac{r_{ik} - r_{kf}}{r_{kg} - r_{kf}}$$

The tables below outline the analysis:

Table S9: Jaksland analysis parameters.

Separation process	Feasibility ratio, r_kf	Good ratio, r_kg	Property
Absorption	1.11	2.18	Solubility
Crystallisation	1.2	1.27	Melting point
Distillation	1.01	1.02	Boiling point
Flash	1.23	1.4	Boiling point
L/L extraction	1	2	L/L solubility
Liquid membranes	1.02	1.08	Molar volume
Microfiltration	1.9	2.4	Molecular weight

Due to the limitations of modelling software and lack of experimental data, membranes and microfiltration were not considered. Carbon dioxide is the only gas in the system, hence some of its properties were omitted.

Table S10: Component properties used in Jaksland analysis.

Component	Melting point / K	Boiling point / K	Molar volume / l/kmol	Molecular weight / kg/kmol
Water	273.15	373.15	18.03	18.015
Hydrogen peroxide	272.725	423.35	30.36	34.015
Limonene oxide	265.33	461.74	163	152.236
Carbon dioxide	216.58	194.70	-	-
Limonene	198.8	450.60	162	136.237
Methanol	175.47	337.85	40.45	31.0422

Table S11: Jaksland analysis calculated ratios.

Component pair	Melting point ratio	Boiling point ratio	Molar volume ratio	Molecular weight ratio
Water / hydrogen peroxide	1.001	1.135	1.699	1.888
Water / limonene oxide	1.029	1.237	9.040	8.451
Water / limonene	1.374	1.208	8.985	7.562
Hydrogen peroxide / limonene oxide	1.028	1.091	5.322	4.476
Hydrogen peroxide / limonene	1.372	1.064	5.289	4.005
Limonene oxide / carbon dioxide	1.225	2.372	-	-
Limonene oxide / limonene	1.335	1.025	1.006	1.117
Limonene oxide / methanol	1.512	1.367	4.030	4.751
Carbon dioxide / limonene	1.089	2.314	-	-
Carbon dioxide / methanol	1.235	1.735	-	-
Limonene / methanol	1.133	1.334	4.005	4.252

Table S12: Jaksland analysis results (1).

Component pair	Crystallisation	Distillation	Flash	Liquid membrane	microfiltration
Water / hydrogen peroxide	Not feasible	Good	Not feasible	Good	Not feasible
Water / limonene oxide	Not feasible	Good	Feasible	Good	Good
Water / limonene	Good	Good	Not feasible	Good	Good
Hydrogen peroxide / limonene oxide	Not feasible	Good	Not feasible	Good	Good
Hydrogen peroxide / limonene	Good	Good	Not feasible	Good	Good
Limonene oxide / carbon dioxide	Feasible	Good	Good	-	-
Limonene oxide / limonene	Good	Good	Not feasible	Not feasible	Not feasible
Limonene oxide / methanol	Good	Good	Feasible	Good	Good

Carbon dioxide / limonene	Not feasible	Good	Good	-	-
Carbon dioxide / methanol	Feasible	Good	Good	-	-
Limonene / methanol	Not feasible	Good	Feasible	Good	Good

Table S13: Jaksland analysis results (2).

Component pair	Best separation method	Second best separation method
Water / H_2O_2	Distillation	Liquid membrane
Water / Limonene	Liquid membrane	Distillation
Water / Limonene oxide	Liquid membrane	Distillation
H_2O_2 / Limonene	Liquid membrane	Distillation
H_2O_2 / Limonene oxide	Liquid membrane	Distillation
Limonene / Limonene oxide	Crystallisation	Distillation
Limonene / CO_2	Distillation	Flash
Limonene / Methanol	Liquid membrane	Distillation
Limonene oxide / CO_2	Distillation	Flash
Limonene oxide / Methanol	Liquid membrane	Distillation
CO_2 / Methanol	Distillation	Flash

SI-10b Separation sequencing using Douglas heuristics

Separation sequence 1

- General structure of separation sequence
  The effluent from R1 is liquid, so only liquid separation systems need to be considered for this first separation sequence.
- Remove most plentiful first
  To reduce the size of process units, the most plentiful inert component should be removed before the reactor. Therefore, water is removed in a distillation column prior to R1.
- Remove corrosive and reacting chemicals
  H_2O_2 acts as an oxidant and a reactant in Reaction 1. Therefore, it is removed in the first column after the R1 to minimise the oxidation of process equipment and reaction within the columns.
- Difficult separations last
  The separation of limonene and limonene oxide is the hardest separation in the system due to their similar boiling points. This separation is performed last in this sequence to reduce the reboiler and condenser duties of the columns.

Separation sequence 2

- General structure of separation sequence
  Since the R2 effluent is a two-phase mixture, the reactor should be used as a phase splitter (i.e. a flash vessel after the reactor). This first flash vessel separates CO_2 gas from the reactor effluent, allowing it to be recycled back to R2.
- Difficult separations last
  According to the Jacksland analysis, the most difficult separation in this sequence is the separation of methanol from limonene oxide, since the other separations are relatively easy phase splits. To reduce the heat duties and process equipment size, this separation was performed last.

SI-11 Outputs from orange juice plant

Outputs from orange juice processing plant, according to the literature:

Table S14: Orange juice process outputs.

Output	Annual production [tonnes/a]	Shares in annual turnover [%]
NFC orange juice	40630	85
Animal feed (citrus peel)	9711	7
Animal feed (raw citrus peel)	4405	0
Aseptic orange pulp	1580	5
Essential Oils	198	2
D-Limonene	66.5	1
Total	56590.5	100

Converting the above table for use in this study, assuming:

- All animal feed and aseptic pulp can be categorised as citrus waste.
- Account for water to make citrus waste up to ~50% mass of orange.
- Essential oils are 100% limonene (91-97%).

Table S15: Outputs normalised to 1 kg limonene.

Output	Annual production [tonnes/a]	Normalised to 1 kg limonene	Shares in annual turnover [%]
NFC orange juice	40630.0	153.6	85

Limonene (essential oils + lim)	264.5	1.0	3
Citrus waste + water	15696.0<br>28740.0+<br>44436.0	168.0	12
Total	85330.5	322.6	100

SI-12 Carbon sequestration

The molecular weight of the PLC monomer is 196 g/mol.
This means that there are about 5.1 moles of PLC monomer per kg.
There are 11 carbon atoms per monomer of PLC, so 56.1 moles of carbon per kg PLC.
Multiplying 56.1 moles C by the ratio 44/12 MW(CO2/C) gives 205.8 moles CO_2eq per kg PLC.
Multiplying by the MW of CO_2 and converting to kg yields 9.05 kg CO_2eq per kg PLC.

SI-13 Allocation factors

Mass Allocation

Example calculation for limonene:

$$Mass\ Allocation = \frac{Mass\ Limonene}{Total\ Mass\ of\ outputs} = \frac{264.5}{85330.5} = 0.31\%$$

The mass allocations applied are presented in table [x]:

Table S16: Mass allocations.

Output	Mass Allocation (%)
Limonene	0.3
Orange Juice	47.7
Citrus Waste	52.0

Economic Allocation

Example calculation for limonene:

$$Economic\ Allocation = \frac{Share\ of\ turnover\ Limonene}{100} = \frac{3}{100} = 3\%$$

The economic allocations are presented in table [x]:

Table S17: Economic allocation.

Output	Economic Allocation (%)
Limonene	3
Orange Juice	85
Citrus Waste	12

Energy Allocation

Energy allocation calculations make use of the following equation:

$$e_i = \frac{m_i LHV_i}{\sum_i m_i LHV_i}$$

This equation makes use of the mass allocations (m_i) and the lower heating value (LHV_i) of the output components (i). The mass allocations were calculated previously, and the lower heating values are obtained from literature.

The economic allocations and relevant data are presented in table [x]:

Table S18: Energy allocation method.

Output	Mass allocation (%)	LHV (MJ/kg)	$m_i LHV_i$ (MJ/kg)	Energy allocation (%)
Limonene	0.3	42.9	12.9	7.8
Orange juice	47.7	1.83	87.3	52.5
Citrus waste	52.0	1.27	66.0	39.7

Sample calculation for the energy allocation of limonene:

$$e_{lim} = \frac{m_{lim} LHV_{lim}}{\sum_i m_i LHV_i}$$
$$e_{lim} = \frac{12.9}{166.2} = 7.8\%$$

In the case of limonene, only the higher heating value (HHV) is available in literature so it is necessary to convert this to the LHV using the following calculation.

$$LHV = HHV - m_{H_2O} h_{l,H_2O}$$

The LHV is the HHV minus the latent heat of vapourisation of the water vapour formed during combustion. The sample calculation for limonene is:

$$LHV_{lim} = HHV_{lim} - m_{H_2O} h_{l,H_2O}$$
$$LHV_{lim} = 45.3 - 1.057 \times 2.3$$
$$LHV_{lim} = 42.9\ MJ/kg$$

The HHV of 45.3MJ/kg is taken as average of Aspen data and literature (Pourbafrani et al., 2013). 2.3MJ/kg is the latent heat of vapourisation of water (Datt, 2011). The mass of 1.057kg corresponds to the mass of water produced from the complete combustion of 1kg of limonene, calculated in the following way:

$$C_{10}H_{16} + 14O_2 \rightarrow 10CO_2 + 8H_2O$$

1 mol of limonene ($C_{10}H_{16}$) weighs 136.2g and produces 8 moles of water, weighing 144g total. Therefore 1 kg of limonene will produce 1.057kg of water.

240mL of Orange juice has a calorific content of 110 calories according to Florida Department of Citrus. This can be converted to SI units to obtain the 1.83MJ/kg used in calculations.

For the citrus waste, the LHV was calculated in the following way:

$$LHV_{citrus\,waste}(1 - m_w) \times LHV_{Dry\,biomass} - m_w \times h_{l,w}$$

The moisture content (m_w) of the citrus waste is about 82%. The dry fraction of citrus waste is taken as dry biomass, which has an LHV of 18MJ/kg (Brilman, 2017). The heat of vapourisation of the moisture content can then be subtracted to give the LHV of the wet citrus waste.

$$LHV_{citrus\,waste} = (1 - 0.82) \times 18 - 0.82 \times 2.3$$
$$LHV_{citrus\,waste} = 1.27 MJ/kg$$

SI-14 Inputs to orange juice plant

The inputs to the orange juice process are taken from literature (Jungbluth, 2017) and normalised to 1kg of limonene as shown in table [x].

Table S19: Inputs to orange juice process.

Input	Unit	Amount per L orange juice	Normalised to 1 kg limonene
Oranges	kg	2.29	335.66
Electricity	kWh	0.15	21.99
Natural gas	MJ	0.68	99.67
Tap water	m^3	0.0051	0.75
Detergents (Soda 30-50%)	kg	0.0089	1.30
Detergent nitric acid	kg	0.0003	0.04

SI-15 PLC process inputs

Energy and utility requirements

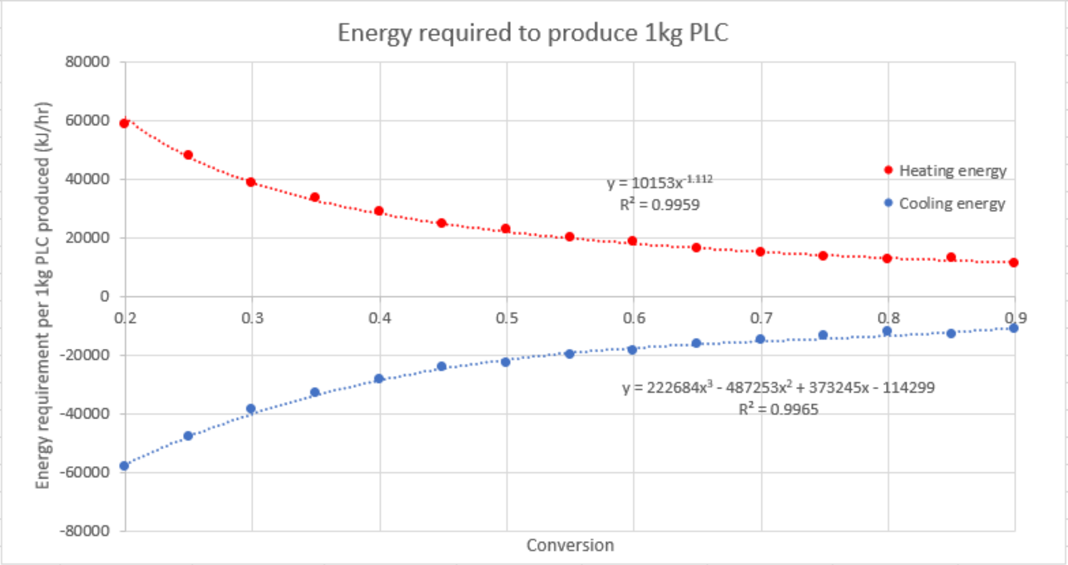

Figure S7: Polynomial regression for PLC process energy requirement.

As the conversion is varied within Aspen, the flowrate of the PLC product stream changes. This is because the process has been optimised relative to a specific optimum conversion and so the operating conditions may be suboptimal at other conversions. This is acceptable from a process/economic point of view, but this report also considers the environmental impact of varying the conversion. To achieve this, the input/output data is normalised relative to the production of 1kg of PLC.

As expected, the energy requirements for the process are reduced as the conversion is increased. This is because the majority of the energy demand is from the separation system. As more unreacted components are recycled in the process, the load on the separation sequence is increased, resulting in higher condenser and reboiler duties. Economies of scale comes into play as the conversion reaches about 0.8; the decrease in energy requirement as a result of increased conversion becomes less dramatic.

The trendlines fits the data with good accuracy (R^2 > 0.98) and therefore can be used to model energy requirement as a function of the conversion. Since the heating requirement will be supplied by burning natural gas, this model can be put directly into SimaPro. The cooling requirement, however, will be supplied by cooling water, so a conversion to cooling water load is required as shown in figure [x].

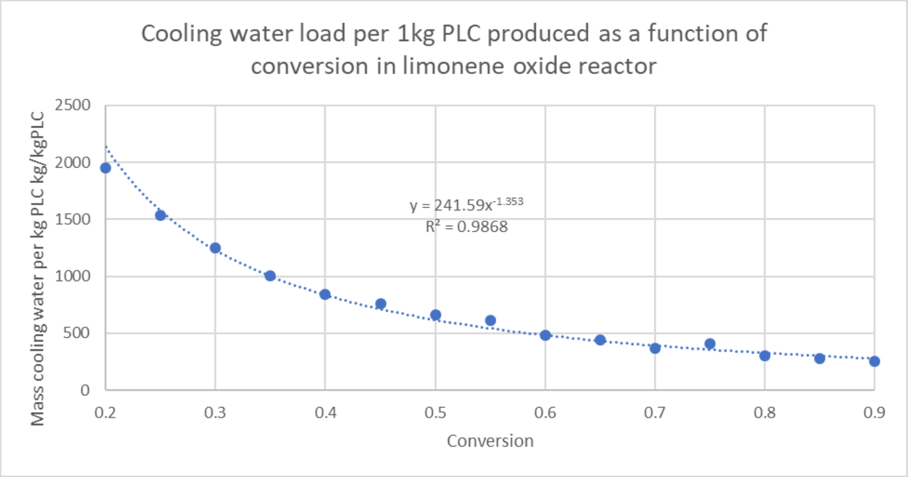

Figure S8: Polynomial regression for PLC cooling water required.

Cooling duty is converting to a cooling water load using equation [x]:

$$\dot{Q} = \dot{m}C_p\Delta T$$

Where $\dot{Q}$ is the cooling energy requirement [kJ/hr], $\dot{m}$ is the mass flow of cooling water [kg/hr], C_p is the specific heat capacity of water [kJ/kgK] and ΔT is the maximum allowable change in temperature of the cooling water [K].

The trendline has a good fit (R^2 = 0.9868) and so this can be used in SimaPro to model the cooling water load requirement as a function of conversion in the limonene oxide reactor.

Component inputs

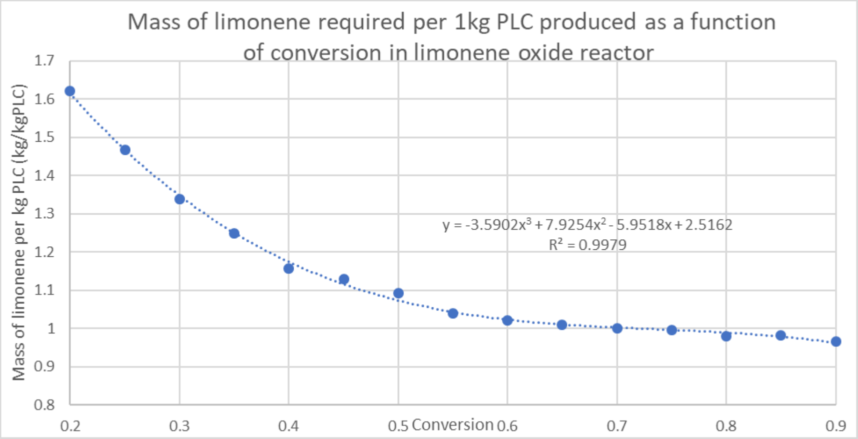

Figure S9: Polynomial regression for limonene input.

As the conversion is increased, the mass of limonene required approaches the stoichiometric ratio of limonene to PLC. The trendline fits the data very well (R^2 = 0.9979) and so this model can be put into SimaPro to determine the amount of limonene to be processed per 1kg of PLC to be produced, as a function of conversion in the limonene oxide reactor. The limonene requirement takes into account the limonene extraction from the citrus waste that was modelled earlier in SimaPro. As the conversion is decreased, the limonene required increases along with all the environmental burdens that are associated with producing the limonene. Therefore, at this stage we can predict that the most environmentally sustainable operation will be achieved for the greatest attainable conversion in the limonene oxide reactor.

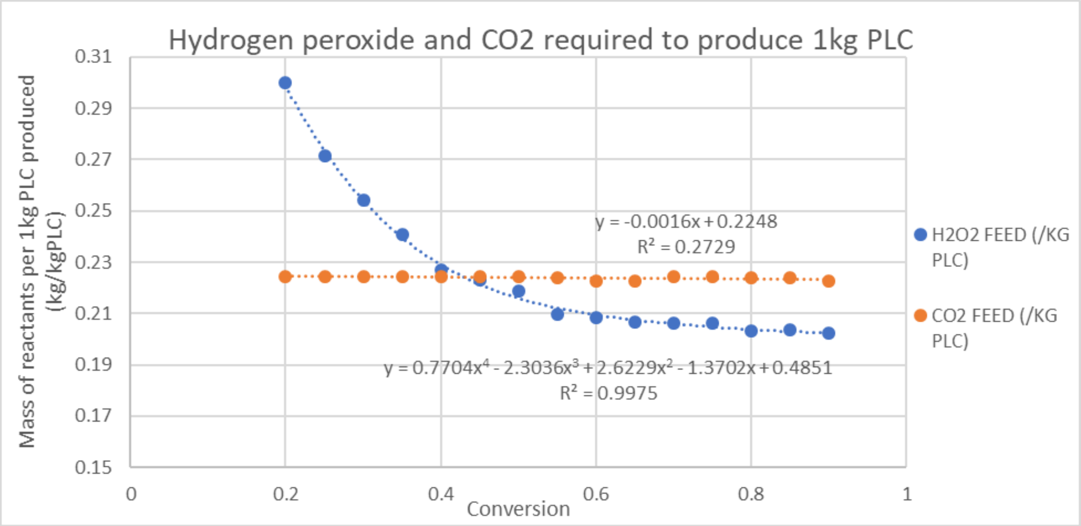

Figure S10: Polynomial regression for hydrogen peroxide and CO2 inputs.

The hydrogen peroxide requirement follows a similar trend to limonene requirement whereas the CO_2 required remains relatively constant as conversion is varied. This is because the CO_2 is not involved in the limonene oxide reaction and only depends on the PLC production rate. Because of this, CO_2 requirement can be modelled as directly proportional to PLC production rate. The hydrogen peroxide in this model is pure, but SimaPro uses H_2O_2 in 50% solution. Because of this the coefficients must be multiplied by 2 when inputting to SimaPro.

SI-16 Energy from PLC waste

The amount of energy produced per mole of each component is summarised in table below.

Table S20: Electricity and thermal energy produced per mol of component processed by AD/CHP.

	Limonene	Limonene oxide	Methanol
COD (g_{o2}/mol)	448	432	48
Volume biogas (m^3)	0.272	0.262	0.019
Total energy (kJ/mol)	3920	3780	420
Electricity (kJ/mol)	1176	1134	126
Heat (kJ/mol)	1960	1890	210
Wasted (kJ/mol)	784	756	84.0

Example calculation of how the COD of limonene oxide is obtained:

Limonene oxide has the chemical formula $C_{10}H_{16}O$. Writing this as $C_nH_aO_b$ where n is the number of carbon atoms, a is the number of hydrogen atoms, and b is the number of oxygen atoms, the following equation can then be used to obtain the theoretical COD.

$$COD = \left(n + \frac{a}{4} - \frac{b}{2}\right) \times 32g\ O_2$$

$$COD = \left(10 + \frac{16}{4} - \frac{1}{2}\right) \times 32g\ O_2 = 432g_{O2}/mol$$

This COD can then be converted to a biogas equivalent via the following reaction:

$$CH_4 + 2O_2 \rightarrow CO_2 + 2H_2O$$

In the above reaction, each mole of methane consumes two moles of oxygen. Therefore, 1g of COD destruction is equivalent to 0.35L methane at standard conditions (Speece, 1996). A study carried out by Guo et al. (2013), on a commercial AD plant in the UK, showed that the electricity conversion efficiency is approximately 1.2kWh/m^3 biogas. This electricity conversion is assumed to be about 30% of the biogas calorific value, and 50% of the energy is recovered as thermal energy (with 20% wasted to environment).

Due to the way in which the limonene concentration within the waste streams decreases with increasing conversion, the total amount of energy decreases as shown in figure below.

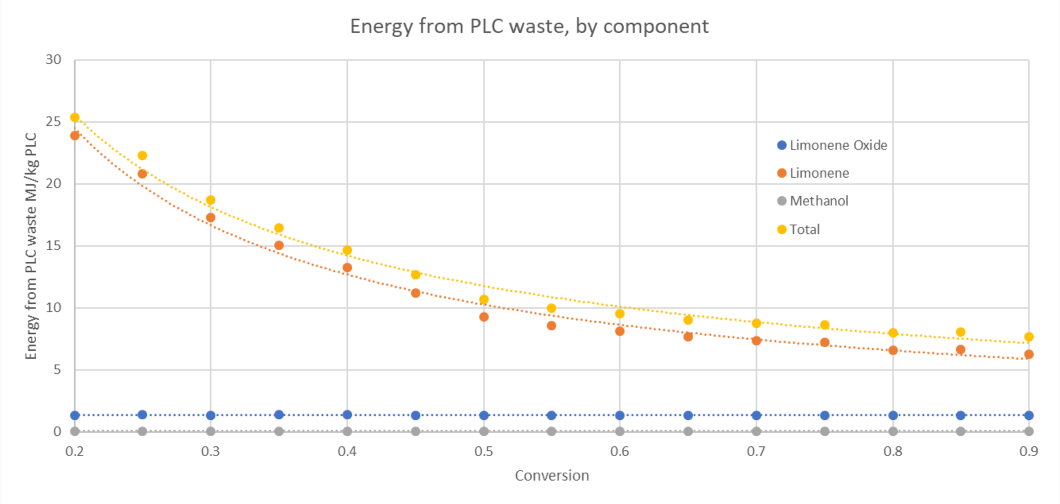

Figure S11: Energy recovered as a function of conversion in epoxidation reactor.

As the conversion in the limonene oxide reactor increases, and therefore the amount of limonene fed decreases, the amount of energy recovered also decreases. Since limonene makes up 40-50% of the PLC waste stream, and due to its high COD, energy from limonene dictates the trend of total energy recoverable from the stream.

SI-17 SimaPro impact assessment raw data

Figure 12

Table S21: Raw data for Figure 12.

x	Human Health (DALY)	Ecosystems (species.yr)	Resources (USD2013)
0.2	1.31E-05	7.44E-08	0.102674
0.3	9.78E-06	5.52E-08	0.086967
0.4	8.14E-06	4.56E-08	0.077874
0.5	7.24E-06	4.04E-08	0.073033
0.6	6.74E-06	3.74E-08	0.070636
0.7	6.44E-06	3.56E-08	0.069407
0.8	6.23E-06	3.44E-08	0.068596
0.9	6.06E-06	3.34E-08	0.067977
1	5.92E-06	3.24E-08	0.067851

Figure 13

Table S22: Raw data for Figure 13.

x	Human Health (DALY)	Ecosystems (species.yr)	Resources (USD2013)
0.2	6.43E-07	7.62E-10	0.051505
0.3	8.25E-07	2.09E-09	0.050087
0.4	9.97E-07	3.25E-09	0.048465
0.5	1.20E-06	4.52E-09	0.048147
0.6	1.41E-06	5.81E-09	0.048705
0.7	1.61E-06	6.98E-09	0.049528
0.8	1.77E-06	7.96E-09	0.050255
0.9	1.91E-06	8.78E-09	0.050927
1	2.08E-06	9.65E-09	0.052051

Figure 14

Table S23: Raw data for Figure 14.

Impact category	Unit	PLC BR	PLC No WRRS	PLC Spain	PS
Climate change	kg CO2 eq	-8.59410E+00	-8.41658E+00	-8.6565456	3.48926E+00
Ozone depletion	kg CFC-11 eq	4.27092E-08	6.22886E-08	3.81E-08	4.08E-09
Terrestrial acidification	kg SO2 eq	3.80695E-03	3.86282E-03	0.00293287	1.03206E-02
Freshwater eutrophication	kg P eq	4.50910E-05	4.40560E-05	4.45E-05	2.61112E-05
Marine eutrophication	kg N eq	1.59927E-03	1.55304E-03	0.00158406	2.24696E-04
Photochemical oxidant formation	kg NMVOC	1.53453E-03	1.70430E-03	0.00114766	9.62206E-03
Particulate matter formation	kg PM10 eq	9.22575E-04	9.55757E-04	0.00066931	3.25886E-03
Water depletion	m3	4.32319E-01	4.31880E-01	0.42771825	1.40128E-01
Fossil depletion	kg oil eq	1.53530E-01	2.04826E-01	0.12826374	1.87007E+00

Figure 15

Table S24: Raw data for Figure 15.

Impact category	Unit	PLC	Limonene	Hydrogen peroxide	Carbon sequested	WRRS
Climate change	kg CO2 eq	0	0.012797	0.050874	-0.98589	-0.01411
Ozone depletion	kg CFC-11 eq	0	9.63E-02	0.903743	0	-0.24888

Terrestrial acidification	kg SO2 eq	0	6.54E-01	0.345646	0	-0.0255
Freshwater eutrophication	kg P eq	0	8.39E-01	0.161171	0	-0.00326
Marine eutrophication	kg N eq	0	9.70E-01	0.029779	0	-0.00206
Photochemical oxidant formation	kg NMVOC	0	3.30E-01	0.669536	0	-0.0804
Particulate matter formation	kg PM10 eq	0	4.99E-01	0.501346	0	-0.0364
Water depletion	m3	0.75528	5.92E-02	0.185489	0	-0.00066
Fossil depletion	kg oil eq	0	8.88E-02	0.911234	0	-0.19464

Figure 16

Table S25: Raw data for Figure 16.

Impact category	Unit	Total	Limonene	Orange, processing grade {BR}| orange production, processing grade | APOS, S	Sodium hydroxide, 50% in H2O, production mix, at plant/RER S	Citrus waste treatment	Electricity, high voltage, production BR, at grid/BR S	Heat, natural gas, at industrial furnace >100kW/RER S	Nitric acid, 50% in H2O, at plant/RER S
Climate change	kg CO2 eq	0.11894	0	0.130872	0.004543	-0.05429	0.015249	0.022172	0.000398
Ozone depletion	kg CFC-11 eq	5.52E-09	0	7.35E-09	2.75E-10	-5.91E-09	6.13E-10	3.18E-09	1.30E-11
Terrestrial acidification	kg SO2 eq	0.002588	0	0.00258	1.94E-05	-4.18E-05	1.33E-05	1.59E-05	1.35E-06
Freshwater eutrophication	kg P eq	3.84E-05	0	3.84E-05	8.27E-08	-6.18E-08	2.96E-08	1.81E-08	8.48E-10
Marine eutrophication	kg N eq	0.001574	0	0.001574	7.46E-07	-1.39E-06	4.19E-07	5.45E-07	7.68E-08
Photochemical oxidant formation	kg NMVOC	0.000558	0	0.000564	1.04E-05	-5.62E-05	1.76E-05	2.16E-05	7.60E-07

Particulate matter formation	kg PM10 eq	0.000483	0	0.000481	6.57E-06	-1.46E-05	5.17E-06	5.21E-06	3.36E-07
Water depletion	m3	0.025944	0.00232895	0.02317	0.00047	-0.00012	7.81E-05	1.60E-05	8.86E-07
Fossil depletion	kg oil eq	0.017095	0	0.02135	0.001238	-0.01552	0.0017	0.008294	3.58E-05

Figure 17

Table S26: Raw data for Figure 17.

Impact category	Unit	PLC (mass)	PLC (econ)	PLC (energy	Polystyrene, general purpose, GPPS, at plant/RER S
Climate change	kg CO2 eq	-8.5944258	-8.2084605	-5.9597936	3.489255
Ozone depletion	kg CFC-11 eq	4.27E-08	6.06E-08	1.65E-07	4.08E-09
Terrestrial acidification	kg SO2 eq	0.003806721	0.012204432	0.061130228	0.01032055
Freshwater eutrophication	kg P eq	4.51E-05	0.000169773	0.00089618	2.61E-05
Marine eutrophication	kg N eq	0.001599264	0.006708002	0.036471953	0.000224696
Photochemical oxidant formation	kg NMVOC	0.001534215	0.003345042	0.013895077	0.009622064
Particulate matter formation	kg PM10 eq	0.000922499	0.002490806	0.011627902	0.003258862
Water depletion	m3	0.43231876	0.51650977	1.0070139	0.14012792
Fossil depletion	kg oil eq	0.15340867	0.20888344	0.53208427	1.8700716

SI-18 First law analysis

The net of the cooling and heating requirements can be calculated and used as an initial estimate of the additional heating or cooling required by the process. At least an additional 600 MJ/hr of cooling was required, however this is likely an underestimate since this analysis does not take into account The Second Law of Thermodynamics, nor the minimum driving force requirement.
